# Mice Lacking *Brinp2* or *Brinp3*, or Both, Exhibit Behaviors Consistent with Neurodevelopmental Disorders

**DOI:** 10.3389/fnbeh.2016.00196

**Published:** 2016-10-25

**Authors:** Susan R. Berkowicz, Travis J. Featherby, James C. Whisstock, Phillip I. Bird

**Affiliations:** ^1^Biomedicine Discovery Institute and Department of Biochemistry and Molecular Biology, Monash UniversityClayton, VIC, Australia; ^2^Melbourne Brain Centre, Florey Neuroscience InstituteParkville, VIC, Australia; ^3^ARC Centre of Excellence in Advanced Molecular Imaging, Monash UniversityClayton, VIC, Australia

**Keywords:** *Brinp2*, *Brinp3*, knock-out mice, ADHD, anxiety, neurodevelopmental disorders

## Abstract

**Background:** *Brinps 1–3*, and Astrotactins (*Astn*) *1 and 2*, are members of the Membrane Attack Complex/Perforin (MACPF) superfamily that are predominantly expressed in the mammalian brain during development. Genetic variation at the human *BRINP2/ASTN1* and *BRINP1/ASTN2* loci has been implicated in neurodevelopmental disorders. We, and others, have previously shown that *Brinp1*^−/−^ mice exhibit behavior reminiscent of autism spectrum disorder (ASD) and attention deficit hyperactivity disorder (ADHD).

**Method:** We created *Brinp2*^−/−^ mice and *Brinp3*^−/−^ mice via the Cre-mediated LoxP system to investigate the effect of gene deletion on anatomy and behavior. Additionally, *Brinp2*^−/−^*Brinp3*^−/−^ double knock-out mice were generated by interbreeding *Brinp2*^−/−^ and *Brinp3*^−/−^ mice. Genomic validation was carried out for each knock-out line, followed by histological, weight and behavioral examination. *Brinp1*^−/−^*Brinp2*^−/−^*Brinp3*^−/−^ triple knock-out mice were also generated by crossing *Brinp2/3* double knock-out mice with previously generated *Brinp1*^−/−^ mice, and examined by weight and histological analysis.

**Results:** *Brinp2*^−/−^ and *Brinp3*^−/−^ mice differ in their behavior: *Brinp2*^−/−^ mice are hyperactive, whereas *Brinp3*^−/−^ mice exhibit marked changes in anxiety-response on the elevated plus maze. *Brinp3*^−/−^ mice also show evidence of altered sociability. Both *Brinp2*^−/−^ and *Brinp3*^−/−^ mice have normal short-term memory, olfactory responses, pre-pulse inhibition, and motor learning. The double knock-out mice show behaviors of *Brinp2*^−/−^ and *Brinp3*^−/−^ mice, without evidence of new or exacerbated phenotypes.

**Conclusion:** *Brinp3* is important in moderation of anxiety, with potential relevance to anxiety disorders. *Brinp2* dysfunction resulting in hyperactivity may be relevant to the association of ADHD with chromosome locus 1q25.2. *Brinp2*^−/−^ and *Brinp3*^−/−^ genes do not compensate in the mammalian brain and likely have distinct molecular or cell-type specific functions.

## Introduction

BMP/RA-inducible neural-specific protein(s) (*Brinps*) are a family of three highly conserved vertebrate genes that are almost exclusively expressed in neurons in the central and peripheral nervous system (Kawano et al., 2004; Terashima et al., 2010). They form part of a larger protein superfamily exemplified by the membrane attack complex/perforin (MACPF) domain. *Brinp2* and *Brinp3* are expressed in differentiated neurons of the neocortex, amygdala, hippocampus, and cerebellum during mammalian brain development, peaking at postnatal day 14 (Kawano et al., 2004). In the adult mouse brain, *Brinp2* and *Brinp3* gene expression is reduced and region-specific expression, unlike the more ubiquitous expression of *Brinp1*. *Brinp2* and *Brinp3* show a partial temporal and regional overlap in expression during development of the mammalian brain (Kawano et al., 2004). High homology between *Brinp2* and *Brinp3* amino acid sequence (70% identity) suggests that these two genes evolved by duplication of a common ancestor (Kawano et al., 2004; Giousoh et al., 2015), and may perform a similar, interchangeable function—to the extent that one may compensate for the other's absence. Furthermore, *Brinp1* is ~50% homologous to *Brinp2* and *Brinp3*, suggesting all three *Brinps* have related roles (Kawano et al., 2004).

*BRINP2, BRINP3*, and the MACPF superfamily member *Astrotactin1* (*ASTN1*) are co-located in humans at chromosome 1q25.2, and in mice (Giousoh et al., 2015). Genetic variations at the 1q25.2 locus are associated with neurodevelopmental disorders, in particular attention deficit hyperactivity disorder (ADHD) (Lesch et al., 2008; Romanos et al., 2008). Two copy number variations (CNV)—one deletion and one duplication—are reported in patients with neurodevelopmental disorders (NDDs) that span the *ASTN1/BRINP2* loci, suggesting that one or both of these genes is responsible for the neuropathology (Lionel et al., 2014). The patient with a CNV gain had anxiety, ASD, learning disability, and motor delay, whereas the patient with the deletion suffered developmental delay and seizures (Lionel et al., 2014). In addition, *BRINP2* has been linked to substance abuse and reward dependence in two genome-wide association studies (Verweij et al., 2010; Drgon et al., 2011). Patients diagnosed with schizophrenia show altered methylation states of *Brinp3* (Numata et al., 2014). Changes in *BRINP3* (*FAM5C*) levels have also been reported to correlate with changes in the neurotransmitter norepinephrine during exercise (Karoly et al., 2012).

In non-NDD diagnoses, overexpression of *BRINP2 (FAM5b) or BRINP3 (FAM5c)* has been implicated in cancer: *BRINP2* is amplified in oral squamous cell carcinoma (Cha et al., 2011), and *BRINP3* is overexpressed in pituitary adenomas (Shorts-Cary et al., 2007). Both genes also show association with coronary heart disease (Connelly et al., 2008; Angelakopoulou et al., 2012). Presentation in similar disease suggests a common molecular function of *BRINP2* and *BRINP3*.

*Brinp1* was the first of the three *Brinp* genes to be studied in mice. *Brinp1*^−/−^ mice generated by our group and others show hyperactivity, decreased body weight, reduced reproductive success, impaired short-term memory, altered anxiety response, and social communication impairments reminiscent of autism spectrum disorder (ASD). In the adult brain, *Brinp1*^−/−^ mice exhibit increased parvalbumin-interneuron density in the neocortex and hippocampus (Kobayashi et al., 2014; Berkowicz et al., 2016).

In this study, the key aims were to determine the effect of loss of *Brinp2* and/or *Brinp3* on mouse anatomy and behavior, and to gain insight into the (potentially overlapping) function of these genes and their relationship with neurodevelopmental disorders. To investigate if compensation by *Brinp2* or *Brinp3* masks a more severe phenotype in the *Brinp1*^−/−^ mice, *Brinp1*^−/−^*Brinp2*^−/−^*Brinp3*^−/−^ (*Brinp1/2/3*) triple knock-out mice were also generated and studied.

## Materials and methods

### Gene targeting

Gene targeting methods were similar to those described for the generation of *Brinp1*^−/−^ mice (Berkowicz et al., 2016). A targeting vector was constructed to alter the *Brinp2* locus in mouse embryonic stem (ES) cells by homologous recombination following the general strategy outlined in Teoh et al. (2014). Separately, a targeting vector was constructed to alter the *Brinp3* locus in ES cells. The vectors were built using bacterial artificial chromosome (BAC) clones RP23-97A3 and RP23-213F2 as the source of *Brinp2* DNA, and BACs RP23-146N23/RP23-301J4 as the source of *Brinp3* DNA. Both the *Brinp2* and *Brinp3* vectors comprised a neomycin transcriptional unit flanked by flippase (Flp) recognition target (FRT) elements placed in intron 3. For each vector, a loxP element was placed in the same intron immediately downstream of the neomycin cassette, while an upstream loxP element was placed in intron 2. Cre-recombinase mediated deletion of exon 3 was designed to result in a frame shift, creating a stop codon in the fourth exon. The *Brinp2* and *Brinp3* targeting constructs were separately electroporated into Bruce 4 C57BL/6-derived embryonic stem (ES) cells, and the targeted clone carrying the targeted allele *Brinp2*^*tm1Pib*^ (MGI:5604614) or *Brinp3*^*tm1Pib*^ (MGI:5604619) were identified by Southern analysis. A correctly targeted clone for each gene was injected into BALB/c blastocysts to generate chimeric mice. Chimeric *Brinp2*^*tm1Pib*^ and chimeric *Brinp3*^*tm1Pib*^ mice were each crossed to C57BL/6 Cre-deleter transgenic mice (Tg(CMV-cre)1Cgn) to remove exon 3 and the neomycin cassette from the targeted allele to produce animals carrying the *Brinp2*^*tm1.1Pib*^ mutation (MGI:5604615) or *Brinp3*^*tm1.1Pib*^ mutation (MGI:5604620). In parallel, chimeric mice were crossed to C57BL/6 Flp-deleter transgenic mice to remove the neomycin cassette only [*Brinp2*^*tm1.2Pib*^ (MGI:5604617); *Brinp3*^*tm1.2Pib*^ (MGI:5604621)]. “Floxed” mice heterozygous for the *Brinp2*^*tm1.1Pib*^ mutation were inter-crossed to generate mice of all three genotypes: *Brinp2*^+/+^ (WT); *Brinp2*^+/*tm1.1Pib*^ (het); and *Brinp2*^*tm1.1Pib*/*tm1.1Pib*^ (*Brinp2*^−/−^). “Floxed” mice heterozygous for the *Brinp3*^*tm1.1Pib*^ mutation were inter-crossed to generate mice of all three genotypes: *Brinp3*^+/+^ (WT); *Brinp3*^+/*tm1.1Pib*^ (het); and *Brinp3*^*tm1.1Pib*/*tm1.1Pib*^ (*Brinp3*^−/−^).

### Genomic analysis

Mice carrying the *Brinp2*^−/−^ and *Brinp3*^−/−^ floxed alleles, along with their respective WT, targeted, and Flpe mouse lines were all verified by Southern analysis. Genomic DNA isolated from the spleen was digested with *Ase*I (*Brinp2)* or *Nde*I (*Brinp3)* and probed with a 500 bp 5′ homology probe. A 3′ homology arm probe was used for blotting of genomic DNA digested with *BglII* (*Brinp2) PvuII* (*Brinp3)*. An internal probe was used to rule out random integration into the genome. Genotyping PCR confirmed absence of the neomycin cassette in floxed animals.

### Breeding *Brinp2/3^−/−^* double knock-out mice

*Brinp2*^−/−^ mice were mated with *Brinp3*^−/−^ mice. The resultant heterozygous offspring were bred, avoiding sibling matings. As both genes are present on chromosome 1q (separated by 11.8 M bp) offspring were screened for a crossover event occurring between the *Brinp2*^*tm1.1Pib*^ allele and the *Brinp3*^*tm1.1Pib*^ allele, resulting in a chromosome with the mutant version of both genes.

The presence of a *Brinp2*^*tm1.1Pib*^ *Brinp3*^*tm1.1Pib*^ chromosome was screened for as a genotyping result showing a mouse homozygous for one floxed gene, and heterozygous for the other—a scenario only possible if a cross-over event has occurred. Mice found to have *Brinp2*^*tm1.1Pib*^ and *Brinp3*^*tm1.1Pib*^ on the same chromosome were bred to generate *Brinp*2/3^−/−^ mice. *Brinp*2/3^−/−^ mice were identified by genotyping PCR, and validated by reverse-transcriptase PCR (RT-PCR).

*Brinp2/3* knock-out mice were maintained as a homozygous colony to prevent recombination re-occurring in order to conserve the *Brinp2/Brinp3*^*tm1PB*^ alleles. A WT line from the same parental origins was maintained in parallel.

### Breeding *Brinp1/2/3^−/−^* triple knock-out mice

Female *Brinp2/3*^−/−^ mice were bred with male *Brinp1*^−/−^ mice, resulting in mice heterozygous for *Brinp1*^*tm1.1Pib*^, *Brinp2*^*tm1.1Pib*^, and *Brinp3*^*tm1.1Pib*^. Triple heterozygous mice were mated to generate *Brinp1/2/3*^−/−^ mice at an expected frequency of 1/16, along with WT littermates. Additionally, male mice of genotype *Brinp1*^+/−^*/*2^−/−^/3^−/−^ were mated with female triple heterozygous mice in an attempt to increase triple knock-out mice frequency. *Brinp1/*2/3^−/−^ mice were identified by genotyping PCR, and validated by RT-PCR.

### RT-PCR

RNA was extracted from the whole brain of WT, het and knock-out mice from each *Brinp* line and reverse transcribed into cDNA (SSIII First Strand Synthesis, Life Sciences). Primers used for *Brinp1* primers were designed to Exon 2: 5′-CTGGGACAGACCAACATGTCTC and Exon 6: 3′-GCTCTCCGTGCTTTGCAGAAGG, to produce a 526 bp WT product or a 336 bp floxed product.

*Brinp2* primers were designed to Exon 2: 5′-wordGGACTGGCTGCTCACAGACCG and Exon 4: 3′-wordGTGCTCTCTCTGTCAATGAAG, to produce a 439 bp WT product or a 247 bp floxed product.

*Brinp3* primers were designed to Exon 2: 5′-wordCCCCTTCGACTGGCTCCTCTC and Exon 5: 3′-wordCCTGTCCGTGTTTCTGTCACC, to produce a 510 bp WT product or a 221 bp floxed product. PCR conditions: 95°C 60 s (95°C 30 s, 61°C 30 s, 72°C 30 s) × 35, 72°C 120 s. RT-PCR products were cut out of a 2% agarose gel and sequenced.

### Animals

C57BL/6 floxed mice and wild type littermates were generated from heterozygous breeders in all studies. Mice were genotyped from tail snips collected at postnatal day 10 (P10). For each knock-out line, mice were housed with mixed genotype littermates; with a maximum of five adults per box. Mice were housed in individually ventilated, sawdust lined Thoren cages, and fed *ad libitum* on Barastoc rodent feed with constant access to water. Mice were maintained on a 12 h light/dark cycle (light 7 a.m.–7 p.m.) and with a controlled room temperature of 18–24°C. Cages and bedding were changed weekly. All breeding and experiments were approved by the Monash University Animal Ethics Committee.

### Weighing

*Brinp2*^−/−^, *Brinp3*^−/−^ *Brinp2/3*^−/−^, *Brinp1/2/3*^−/−^ mice, along with their respective WT littermates were weighed weekly between 3 and 12 weeks.

### Histology

Twenty-five organs per mouse were compared to their respective WT littermates from juvenile (7–8 week old) *Brinp2*^−/−^, *Brinp3*^−/−^, *Brinp2/3*^−/−^, and *Brinp1/2/3*^−/−^ mice. Tissue was prepared as formalin fixed, paraffin wax embedded sections (10 μm). Hematoxylin and Eosin (H&E) staining was carried out by the Australian Phenomics Network (http://www.australianphenomics.org.au/). Histopathological assessments and clinical hematological analysis was performed on two mice per genotype and compared to WT littermates. The same 25 organ types were examined for macromorphological abnormalities as previously described (Berkowicz et al., 2016). For a list of organs examined, refer to the characterization of *Brinp1*^−/−^ mice.

### Reproductive phenotyping

Three breeding pairs per genotype (WT/WT, Triple-Het/Triple-Het, and Triple-Het ♂/*Brinp1*^−/+^2^−/−^3^−/−^ ♂) were set up and monitored over 5 months. Mice were first used as breeders at 7–8 weeks of age. The number of pups were recorded at birth and at weaning (postnatal day 21).

### Behavioral testing

Cohort sizes were 9–12 mice, aged 3–4 months. Where possible, a ratio of 1:1 females/males was tested. Mice were habituated to the testing facility for 1 week, then habituated to the testing room overnight. Mice were tested by experimenters blind to genotype and in random order. Tests were separated by a minimum of 1 day. WT and knock-out mice were tested in the same testing sessions. Lighting conditions were 30 lux for all behavioral tests. Testing arenas were cleaned with Equinade disinfectant (lavender scent) between trials. In all instances, mice had previously been habituated to the disinfectant whilst housed at the testing facility. Behavioral tests were performed in an identical manner to previously described for the behavioral characterization of *Brinp1*^−/−^ mice (Berkowicz et al., 2016), as follows.

### Visual placing test

Mice were lifted by the tail to a height of 15 cm and lowered onto a mesh grid within 1 s, decelerating as the grid approached. The distance of the animal's nose from the grid was measured the moment before the mouse extended its forelimbs toward it. A single trial was performed per animal.

### Olfaction test

Relative levels of sniffing behavior was investigated within an area that contained 2 squares of filter paper (4 × 4 cm), one containing 275 μl of peanut butter (at three dilutions—1:10; 1:100; 1:1000) and one containing 275 μl of water, over a 3 min period. The animals were first acclimatized to the area for a 15 min period.

### Rotarod

Mice were pre-trained on the Rotarod (Ugo Basil) for two initial trials at a constant speed of 4 rpm for 5 min, followed by a third trial accelerating from 4 to 40 rpm over 5 min. Testing was carried out the following day by 4 × 5 min accelerating trials with an inter-trial interval of 30 min.

### Elevated plus maze

Mice were placed on an elevated platform (material: Perspex, color: beige) at a height of 40 cm above the floor. The platform comprised of two open arms and two closed arms (each 4.5 cm wide, 30 cm in length), connected by a central square (6 × 6 cm). The two closed arms were protected by a 15 cm high wall. Mice were placed on the center square and video recorded whilst exploring the maze. Time and frequency of entry into each arm, and average velocity throughout the trial time of 5 min were recorded. Tracking software: Noldus Ethovision 3.0. Frequency of peering down, and entries to the far end of the open arm, were counted manually. Entries into the far ends of the open arms were defined as all feet greater than halfway along the arm.

### Y-maze

Mice were tested in two trials of a Plexiglass Y-maze (material: Perspex, color: gray) with each of the three arms having a distinctive visual cue at the end. Dimensions of each arm were 30 × 10 cm, with a triangle center zone of 10 cm equal sides. Mice received a random association between visual cues and arm location. In Trial 1, a partition blocked off the left arm of the maze. The mouse was placed at the end of the home arm, facing away from the center. The time spent in each of the two available arms over 10 min was recorded. Mice were rested for 2 h. In trial 2, testing was repeated in a second trial with the partition removed and all three arms made accessible. Time in each arm and average velocity was recorded for trial 2 for 10 min. Tracking software: Noldus Ethovision XT 5.0.

### Acoustic startle and pre-pulse inhibition (PPI)

Mice were placed individually inside a Perspex cylinder, closed at both ends. The cylinder was placed upon a platform sensitive to weight displacement, within a sound attenuating box with a background sound level (San Diego Instruments Startle Response System). The background white noise level was set to 70 dB. To measure acoustic startle, a strong 40 ms startle sound was played and startle response was measured by the jumping reflex (<1 s) as weight displacement on the platform. Pre-Pulse Inhibition (PPI) was measured as the percentage reduction in startle response when a non-startling 20 ms pre-pulse of (a) 4 db (b) 8 db (c) 16 db above the 70 dB background sound was played 100 ms prior to the startle sound.

### Three chamber social interaction test

Identical rectangular wire cages were placed in equivalent positions in the left and right chambers of a three-chamber plexiglass box (600 × 400 × 250 mm). Mice were habituated to the empty cages in the left and right chambers (trial 1) and time interacting with each cage was recorded. In trial 2, an unfamiliar C57BL/6J WT sex-matched mouse was introduced to one of the cages, and time interacting with each cage was again recorded. Each trial lasted 10 min, with average velocity recorded for each trial. The mice serving as strangers were habituated to placement under the wire cage for 5 min prior to the test. Mice were tracked using Cleversys Tracking and Topscan software. The interaction zone was defined by the software as an unmarked perimeter zone of 2 cm around the metal cages. Interaction time was defined as nose within the interaction zone. The chambers were cleaned between trials with Equinade disinfectant (lavender scent).

### Statistical analysis

Survival rates of total numbers of mice weaned was analyzed by Chi-square test. Number of surviving mice per litter was analyzed by one-way ANOVA. Postnatal weight, olfaction test, Rotarod, Elevated Plus Maze, and Y-maze performance, and PPI analyzed by repeat measures two-way ANOVA. Vision test, startle response and EPM and Y-maze secondary data was analyzed by Student *t*-test. Habituation trials and social interaction test were analyzed by one-way ANOVA. Average velocity for trial 1 (habituation) and trial 2 of the social interaction test was analyzed by two-way repeat measures ANOVA. Male and female mice were analyzed separately, unless otherwise stated. Behavioral data was represented as the mean ± standard deviation. A value of *p* < 0.05 was used to determine significance.

## Results

### Targeting of *Brinp2* knock-out mice

**Figure 1:**
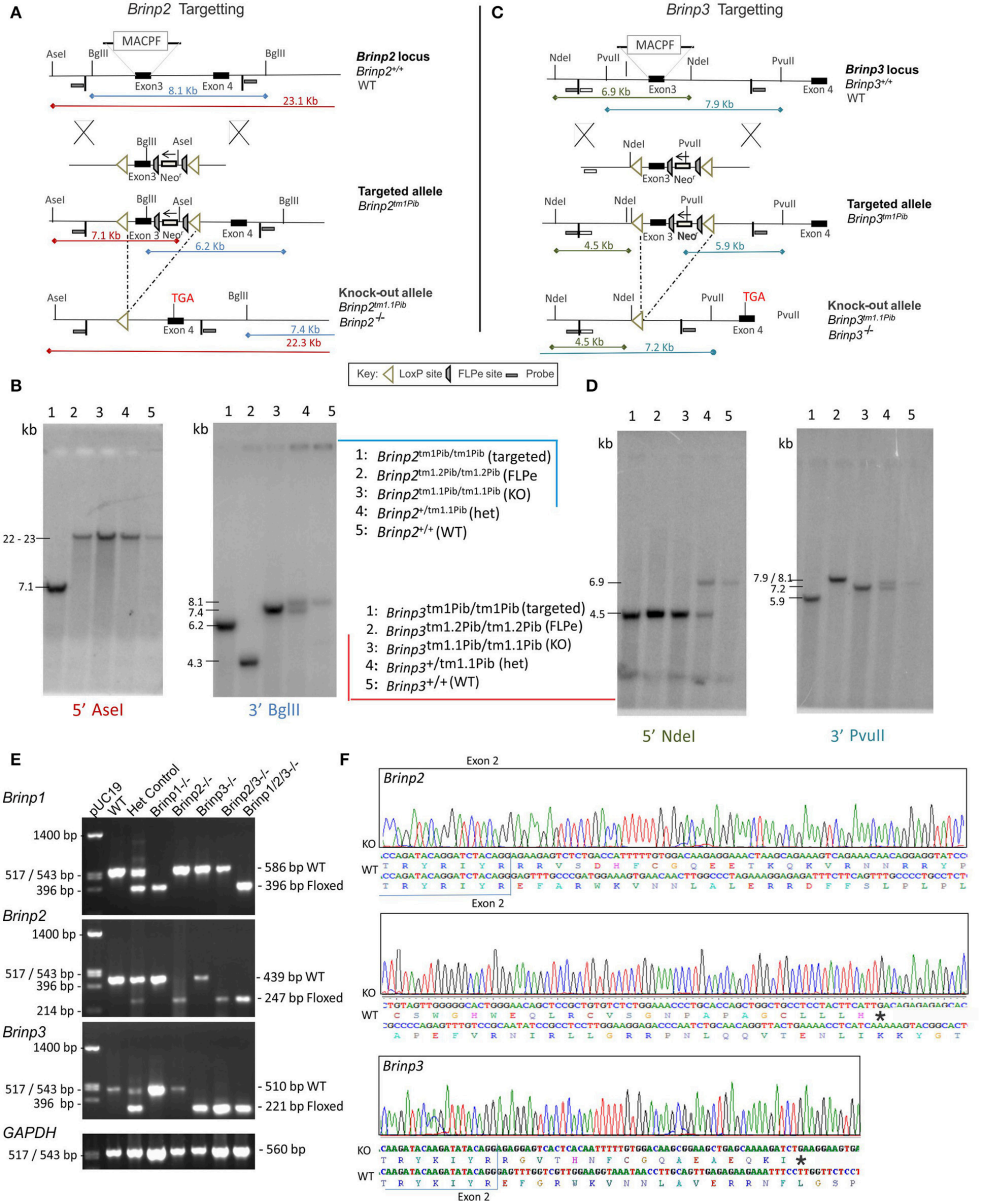
*****Brinp2*** and ***Brinp3*** Targeting. (A)** The *Brinp2* targeting vector was designed with a Neomycin resistance (Neo-r) cassette after exon 3, and FRT sites placed before and after the Neo-r cassette. The 192 bp 3rd exon of *Brinp2* contains the start of the Membrane Attack Complex/Perforin (MACPF) domain. LoxP sites flank exon 3 and the Neo-r cassette. When crossed with a global Cre-deleter mouse line, the recombination of LoxP sites resulted in the deletion of exon 3 and the Neo-r cassette. **(B)** *Brinp2* Southern Blot: splenic genomic DNA was cleaved with PstI and BglII and hybridized to 500 bp genomic DNA probes from the 5′ region (Asel) and 3′ region (Bgl II) of the targeting construct. In wild-type DNA, species of 23.1 kb (Asel) and 8.1 kb (Bgl II) were detected. The 8.1 kb (Bgl II) species was not present in DNA from *Brinp2*^−/−^ mutants, replaced with shorter species 7.4 kb (BglII). **(C)** The *Brinp3* targeting vector was designed with a Neo-r cassette after exon 3, and FRT sites positioned before and after the Neo-r Cassette. The 289 bp 3rd exon of *Brinp3* contains the start of the MACPF domain. LoxP sites flank exon 3 and the Neo-r cassette. When crossed with a global Cre-deleter mouse line, the recombination of LoxP sites resulted in the deletion of exon 3 and the Neo-r cassette. **(D)** *Brinp3* Southern Blot: splenic genomic DNA was cleaved with PstI and BglII and hybridized to 500 bp genomic DNA probes from the 5′ region (Ndel) and 3′ region (PvuII) of the targeting construct. In wild-type DNA, species of 6.9 kb (Ndel) and 7.9 kb (PvuII) were detected. These products were not present in DNA from *Brinp3*^−/−^ mutants, replaced with shorter species of 4.5 kb (Ndel) and 7.2 kb (PvuII). **(E)** Validation of knock-out line using cDNA derived from brain tissue mRNA from WT, heterozygous and *Brinp2*^−/−^ *Brinp3*^−/−^ *Brinp2/3*^−/−^ *Brinp1/2/3*^−/−^ mice. For each genotype, PCR product sizes correspond to the removal of exon3 in the knock-out allele. **(F)** Sequencing of the *Brinp2*^−/−^ and *Brinp3*^−/−^ allele RT-PCR products showed the expected absence of exon 3, and that splicing fuses exons 2 and 4, resulting in a frame shift that introduces a stop codon after 50 (*Brinp2*^−/−^) and 18 (*Brinp3*^−/−^) residues. Sequencing results for *Brinp1*^−/−^ allele can be found in previously published work (Berkowicz et al., 2016).

A conditional *Brinp2* targeted allele (*Brinp2*^*tm1*/*Pib*^) was designed to allow Cre-recombinase-mediated, tissue-specific deletion of *Brinp2* (Figure 1A). Mice lacking *Brinp2* in all tissues were generated by breeding animals carrying the targeted allele with animals expressing Cre-recombinase from the two-cell embryonic stage onwards (global Cre-deleter). Progeny exhibiting deletion of the selection cassette and third exon of *Brinp2* (*Brinp2*^*tm1.1*/*Pib*^) were inter-bred to generate homozygous *Brinp2*^*tm1.1Pib*/*tm1.1Pib*^ (*Brinp2*^−/−^) animals and WT littermates. Correct targeting and deletion of exon 3 was confirmed by Southern analysis (Figure 1B), RT-PCR (Figure 1E), and DNA sequencing of RT-PCR products (Figure 1F). The resultant mRNA lacking exon 3 reflects forced splicing between the intron 2 donor and intron 4 acceptor, fusing exons 2 and 4, and changing the reading frame to introduce a truncating stop codon (Figure 1F). The predicted mutant protein would comprise the cleavable signal peptide (33 aa) and 56 aa of the 750 aa mature BRINP2 protein, and contains no recognizable functional domains. Hence this 56 aa form is missing over 92% of the BRINP2 amino acid sequence, and would be highly unlikely to fold correctly. It is therefore likely degraded shortly after synthesis, which is the generally accepted fate of truncated or misfolded proteins (Hiller et al., 1996).

### Targeting of *Brinp3* knock-out mice

In an identical manner to the *Brinp2* targeted mouse line, a conditional *Brinp3* targeted allele (*Brinp3*^*tm1*/*Pib*^) was designed to allow Cre-recombinase-mediated, tissue-specific deletion of *Brinp3* (Figure 1C). Mice lacking *Brinp3* in all tissues were created by breeding animals carrying the *Brinp3* targeted allele with animals expressing global Cre-deleter mice. Progeny exhibiting deletion of the selection cassette and third exon of *Brinp3* (*Brinp3*^*tm1.1*/*Pib*^) were inter-bred to generate homozygous *Brinp3*^*tm1.1Pib*/*tm1.1Pib*^ (*Brinp3*^−/−^) animals and WT littermates. Deletion of exon 3 was confirmed by Southern analysis (Figure 1D), RT-PCR (Figure 1E), and DNA sequencing of RT-PCR products (Figure 1F). The resultant mRNA lacking exon 3 reflects forced splicing between the intron 2 donor and intron 4 acceptor, fusing exons 2 and 4, and changing the reading frame to introduce a truncating stop codon (Figure 1F). The predicted mutant protein would comprise the cleavable signal peptide (33 aa) and 44 aa of the 733 aa mature BRINP3 protein, and contains no recognizable functional domains. Hence this 44 aa form is missing over 93% of the BRINP3 amino acid sequence, and would be highly unlikely to fold correctly, and therefore again likely degraded shortly after synthesis.

### Generation and validation of multigenic *Brinp* deletion

Validation of *Brinp2/3*^−/−^ and *Brinp1/2/3*^−/−^ mice was performed by RT-PCR of cDNA derived from knock-out mouse brain tissue of each genotype. Only PCR products of sizes corresponding with the exon 3-deleted *Brinp2*^*tm1.1Pib*^ and *Brinp3*^*tm1.1Pib*^ alleles were present for *Brinp2/3*^−/−^ mice, confirming homozygosity of both knock-out alleles (Figure 1E). For the *Brinp1/2/3*^−/−^ mouse cDNA, only PCR products of sizes corresponding with the exon3-deleted *Brinp1*^*tm1.1Pib*^, *Brinp2*^*tm1.1Pib*^, and *Brinp3*^*tm1.1Pib*^ alleles were present, confirming homozygosity of all three knock-out exon-3 deleted alleles (Figure 1E).

### *Brinp2^−/−^* and *Brinp3^−/−^* mice are viable and appear at mendelian frequencies; *Brinp1/2/3^−/−^* mice exhibit poor viability

Litters bred from *Brinp2*^+/−^ or *Brinp3*^+/−^ heterozygous crosses were monitored for survival. Mice were genotyped at age of weaning (day 21). Genotypes were assessed for Mendelian inheritance (25% of total progeny expected to be knock-out mice) as an indicator of normal *in utero* and neonatal viability. *Brinp2*^−/−^ and *Brinp3*^−/−^ mice demonstrated close to Mendelian inheritance for the number of knock-outs surviving to age of weaning (*Brinp2*^−/−^: 9/50 = 22.5%, *Brinp3*^−/−^: 10/41 = 24%) and litter sizes from homozygous breeders were normal (*Brinp2*^−/−^: 6.4 ± 2.9 *SD*), *Brinp3*^−/−^: 6.9 ± 1.9 *SD*). *Brinp2/3*^−/−^ homozygotes also produced litters of standard size (6.0 ± 1.9 *SD*) indicating normal viability.

It proved difficult to generate practical numbers of viable *Brinp1/2/3*^−/−^ progeny due to both the low expected frequency from triple heterozygous matings (1 in 16) and the compromised postnatal viability of pups from mothers carrying the *Brinp1*^−/−^ alleles (see Berkowicz et al., 2016). Three triple-het breeders mated for a total of 5 months produced three triple KO mice at a frequency of 3/51 (5.9%), and therefore viable at close to expected frequency of 1/16 (6.3%), however reduced overall litter survival from triple het breeders meant only 3 out of a total of 84 mice pups born were viable triple knock-out mice (Table 1). To increase the likelihood of generating enough triple knock-out mice for a minimum behavioral cohort of 10 within a similar age, female triple heterozygous breeders were paired with male *Brinp1*^+/−^*/*2^−/−^/3^−/−^ mice. These breeders produced normal litter sizes at birth, but many progeny died before weaning, similar to mice carrying *Brinp1* knock-out allele. The addition of the male mice carrying the *Brinp2/3*^−/−^ mutation resulted in a significant decrease in offspring survival and indicates the absence of *Brinp2 and Brinp3*, when combined with *Brinp1* heterozygosity in the male breeder impacts litter survival (Supplementary Figure 1). These limitations meant that it was not possible to generate a triple knock-out behavioral cohort within an appropriate age range.

**Table 1:** **Reproductive phenotyping: generation of ***Brinp1/2/3***^**−/−**^ triple knock-out mice**.

	**Female/Male**		
	**WT/WT**	**Triple Het/Triple Het**	***Triple Het/Brinp1^−/+2−/−3−/−^***
Days from mating to first litter	25	23	28
Days between litters	37	33	30
Number of litters	14	10	14
Number of pups born (P0)	97	84	84
Number of pups weaned (P21)^***^	76	51	25
% Survival	78	61	30
Number of viable *Brinp1/2/3^−/−^* progeny	n/a	3	2

Three breeders per genotype (WT/WT, Triple-Het/ Triple-Het, Triple-Het ♀/Brinp1^−/+^2^−/−^3^−/−^ ♂ were monitored over 5 months to investigate reproductive rates, postnatal survival and viability of Brinp1/2/3^−/−^ mice. Whilst there were no significant differences in the numbers of pups born at postnatal day 0 (P0): $X_{\left(\text{2},\;n=\text{265}\right)}^{\text{2}}$ = 1.276 p = 0.30, Chi-square test, the survival of mice to age of weaning, postnatal day (P21) was significantly impacted by the presence of Brinp1, Brinp2, and Brinp3 deleted alleles carried by breeders:

*** *$X_{\left(\text{2},\;n=\text{152}\right)}^{\text{2}}$ = 25.67, p < 0.001; Chi-square test*.

### *Brinp2^−/−^, Brinp3^−/−^, Brinp2/3^−/−^*, and *Brinp1/2/3^−/−^* mice exhibit reduced body mass, but normal gross morphology

**Figure 2:**
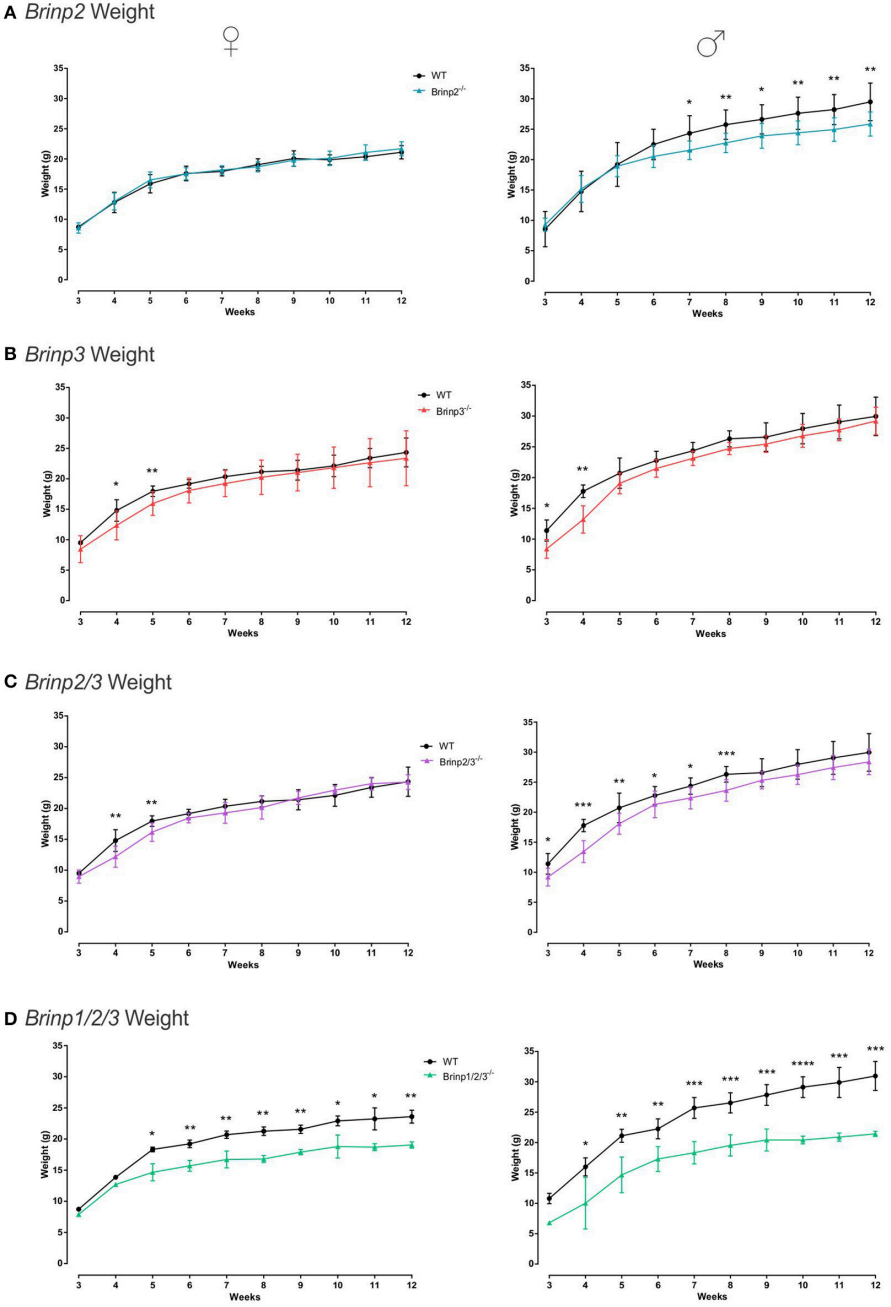
*****Brinp2***, ***Brinp3***, ***Brinp2/3***, and ***Brinp1***/2/3 weights. (A)** *Brinp2*^−/−^ mice weighed from week 3 to 12. Female *Brinp2*^−/−^ mice show normal weight between 3 and 12 weeks by repeat measures two-way ANOVA: *F*_(1, 15)_ = 0.155, *p* = 0.699. Male *Brinp2*^−/−^ mice show a significant reduction in body weight from 6 weeks of age onwards. By repeat measures two-way ANOVA male *Brinp2*^−/−^ mice show a significant interaction of genotype × week: *F*_(9, 117)_ = 5.645, *p* < 0.001. *N* = 8 females, 8 males per genotype. **(B)** *Brinp3*^−/−^ mice weighed from week 3 to 12. Both Male and Female *Brinp3*^−/−^ mice show reduced body mass from weeks 3 to 5, before recovering to near normal weight from week 6 onwards. Female *Brinp3*^−/−^ mice show a significant interaction of genotype × week: *F*_(8, 96)_ = 2.115, *p* = 0.042, repeat measures two-way ANOVA. Male *Brinp3*^−/−^ mice show a significant reduction in body weight to their littermates *F*_(1, 12)_ = 8.602, *p* = 0.013, and a significant genotype × week interaction *F*_(9, 108)_ = 3.919, *p* < 0.001, repeat measures two-way ANOVA. *N* = 7 females, 7 males per genotype. **(C)** *Brinp2/3*^−/−^ mice weighed from week 3 to 12. Female *Brinp2/3*^−/−^ mice show a significant week × genotype interaction effect: *F*_(9, 117)_ = 3.065, *p* = 0.002. Male *Brinp2/3*^−/−^ mice show a significant decrease in body weight, analyzed by repeat measures two-way ANOVA: *F*_(1, 14)_ = 6.228, *p* = 0.026, and a significant interaction effect between week × genotype: *F*_(9, 126)_ = 2.461, *p* = 0.013. *N* = 8 females, 8 males per genotype. **(D)** *Brinp1/2/3*^−/−^ mice weighed from week 3 to 12. Female *Brinp1/2/3*^−/−^ mice show a significant reduction in body weight: *F*_(1, 3)_ = 47.738, *p* = 0.007, with a significant interaction between week × genotype: *F*_(9, 27)_ = 5.736, *p* < 0.001, repeat measures two-way ANOVA, *n* = 3 WT, 2 *Brinp1/2/3*^−/−^ mice. Male *Brinp1/2/3*^−/−^ mice also show a significant decrease in body weight: *F*_(1, 8)_ = 54.708, *p* < 0.001, with a significant interaction between week × genotype: *F*_(8, 64)_ = 3.005, *p* = 0.006, repeat measures two-way ANOVA, *n* = 5 WT, 5 *Brinp1/2/3*^−/−^ mice. Results represented as the mean ± *SD*.

*Brinp2*^−/−^*, Brinp3*^−/−^*, Brinp2/3*^−/−^, and *Brinp1/2/3*^−/−^ mice were weighed weekly from 3 to 12 weeks of age, alongside their respective WT littermates. Knock-out mice from all four lines showed some reduction in body mass. Female *Brinp2*^−/−^ mice displayed normal weight from infant to adult, whereas male *Brinp2*^−/−^ mice showed reduced body mass from 7 weeks onwards, weighing 10% less as adults at week 12 (Figure 2A). *Brinp3*^−/−^ mice weighed less than their WT littermates in the first few weeks of weighing (3–5 weeks) for both males and females, before their weights recovered to that of their WT littermates (Figure 2B). Female *Brinp2/3*^−/−^ mice were smaller in the first few weeks, before recovering to a weight similar to that of WT. Male *Brinp2/3*^−/−^ mice weighed less than WT controls as adults (Figure 2C). Overall, *Brinp2/3*^−/−^ mice show a trend in body mass reduction that reflects a combination of the *Brinp2*^−/−^ and *Brinp3*^−/−^ mice weight profiles.

Female and male *Brinp1/2/3*^−/−^ mice weighed significantly less than WT littermates, and less than *Brinp2*^−/−^, *Brinp3*^−/−^, or *Brinp2/3*^−/−^ mice (Figure 2D). Triple *Brinp* knock-out mice also weighed less the *Brinp1*^−/−^ mice (Berkowicz et al., 2016), indicating a cumulative effect of the triple gene knock-out on body mass.

A full histological examination of juvenile mice for *Brinp2*^−/−^*, Brinp3*^−/−^*, Brinp2/3*^−/−^, and *Brinp1/2/3*^−/−^ mice at 7–8 weeks of age showed normal organ development (25 organs examined) including normal structures in the brain and spinal cord. In all cases, brains appeared symmetrical, with normal myelination, and no ventricular dilation observed (data not shown).

### *Brinp2^−/−^, Brinp3^−/−^*, and *Brinp2/3^−/−^* mouse behavior

To evaluate the effect of *Brinp2* and *Brinp3* loss on neurological function, the behavior of the single and double knock-out lines were assessed. In an initial screen, *Brinp2*^−/−^ and *Brinp3*^−/−^ and *Brinp2/3*^−/−^ mice showed normal auditory, visual, and olfactory capabilities (Supplementary Figures 2A,B). Mice showed no impairments in motor co-ordination on the Rotarod (Supplementary Figures 3A–C), with female *Brinp2*^−/−^ mice showing improved performance on this test.

### *Brinp3^−/−^* knock-out mice exhibit marked changes in exploratory behavior

To examine whether anxiety was affected in the knock-out mice, animals were allowed to freely explore an elevated plus maze (EPM) for 5 min. Mice normally show a species-typical preference for the walled closed arms, and show some hesitance to enter the more exposed open arms. Mice that show a high level of reluctance to approach the open arm are interpreted as having an anxiety-like phenotype.

**Figure 3:**
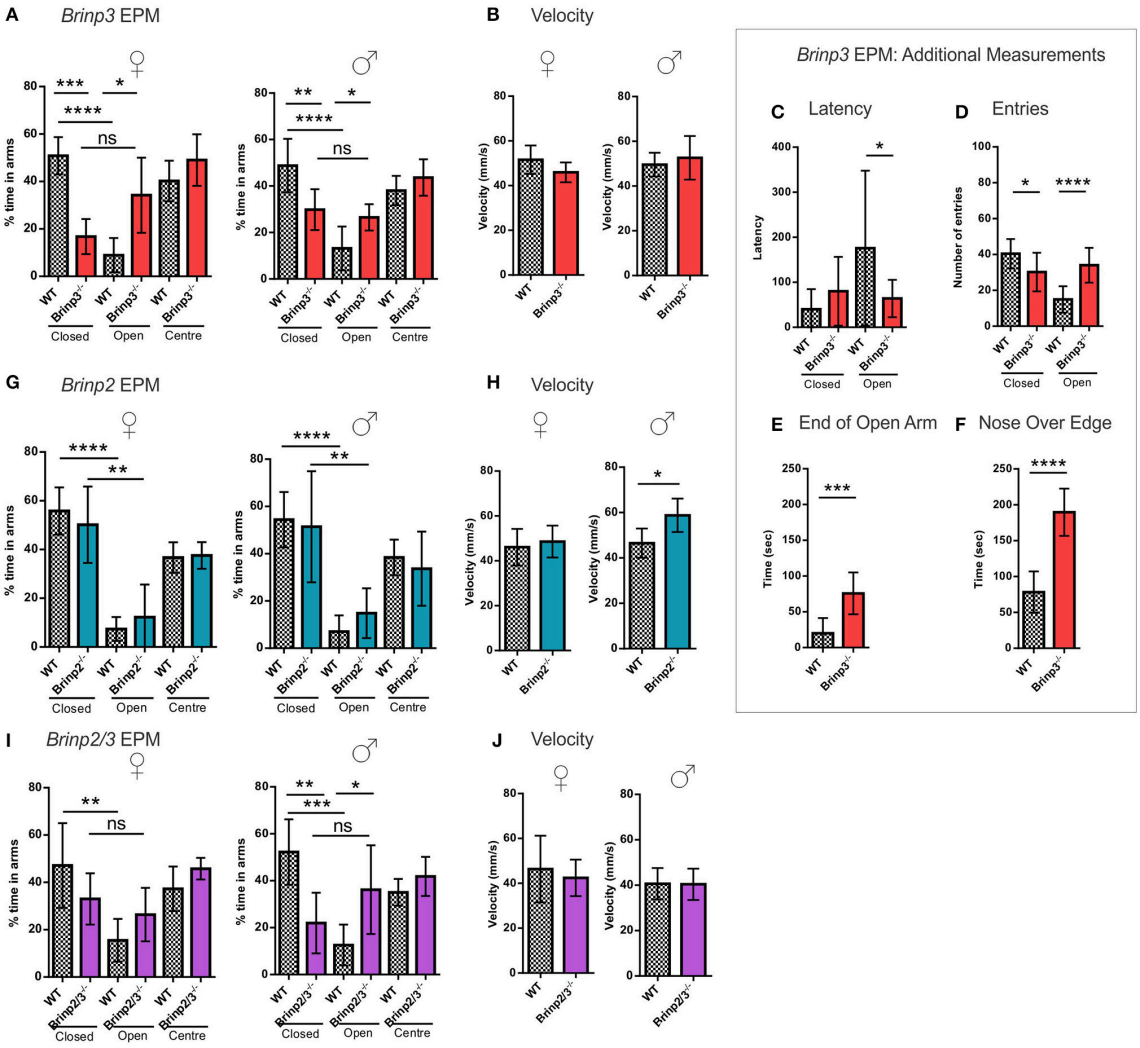
***Brinp3*^−/−^ and *Brinp2/3*^−/−^ mice show increased exploration of the Elevated Plus Maze**. **(A)** *Brinp3*^−/−^ mice spend more time in the open arms relative to the closed arms of the maze, indicating reduced anxiety. Analysis by repeat measure ANOVA shows a genotype × arm interaction: Female: *F*_(2, 16)_ = 15.359, *p* <0.001, male: *F*_(2, 24)_ = 4.407, *p* = 0.023. *N* = 5 female, 7 male mice per genotype. **(B)** Normal *Brinp3*^−/−^ mice average velocity during EPM testing, female: *t*_(8)_ = 1.629, *p* = 0.142, male: *t*_(12)_ = 0.731, *p* = 0.479, Student’s t-test. **(C)** *Brinp3*^−/−^ mice show a significantly reduced latency to enter the open arm: *t*_(22)_ = 2.187, *p* = 0.040, unpaired student *t*-test. **(D)** *Brinp3*^−/−^ show a significantly reduced number of entries into the closed arm: *t*_(21)_ = 2.575, *p* = 0.018 and a significant increase into the open arm: *t*_(21)_ = 5.334, *p* <0.0001, unpaired Student’s *t*-test. **(E)** *Brinp3*^−/−^ mice spend significantly more time peering over the edge of the open arms compared to WT littermates, *t*_(14)_ = 7.190, *p* <0.0001, Student’s *t*-test. **(F)** *Brinp3*^−/−^ mice spend significantly more time at the end of the open arms (defined as >50% of arm length from center), *t*_(14)_ = 4.346, *p* = 0.0007, unpaired Student’s *t*-test. **(G)** No significant effect on arm duration for *Brinp2*^−/−^ mice, female: *F*_(2, 20)_ = 0.552, *p* = 0.584, male: *F*_(2, 20)_ = 0.483, *p* = 0.624, repeat measures two-way ANOVA. *N* = 6 female, 6 male mice per genotype. **(H)** Male *Brinp2*^−/−^ mice show increased average velocity during EPM testing, female: *t*_(10)_ = 0.571, *p* = 0.581, male: *t*_(9)_ = 2.95, *p* = 0.016, Student’s t-test. **(I)** *Brinp2/3*^−/−^ mice: Analysis by repeat measure ANOVA shows a genotype × arm interaction for *Brinp2/3*^−/−^ male mice for percentage time in each arm: Female: *F*_(2, 16)_ = 1.762, *p* = 0.203, male: *F*_(2, 20)_ = 4.571, *p* = 0.023. *N* = 5 female, 6 male mice per genotype. **(J)** Normal *Brinp2/3*^−/−^ mice average velocity during EPM testing, female: *t*_(10)_ = 0.523, *p* = 0.616, male: *t*_(10)_ = 0.055, *p* = 0.958, Student’s *t*-test. ns = not significant. ^*^p <0.05, ^**^p <0.01, ^***^p <0.001, ^****^p <0.0001. Female and male data was combined for graphs **(C–F)**. Results represented as the mean ± SD.

A pronounced phenotype was exhibited by male and female *Brinp3*^−/−^ mice, which spent significantly longer on the open arms and center square, and less time in the closed arms (Figure 3A), indicating reduced anxiety levels. The velocity of *Brinp3*^−/−^ mice was normal for this test, ruling out hyperactivity (Figure 3B). *Brinp3*^−/−^ mice showed a reduced latency time to enter the open arm of the EPM (Figure 3C), and increased number of entries into the open arms (Figure 3D). Additionally, *Brinp3*^−/−^ mice showed prolonged exploration time at the exposed ends of the open arms (Figure 3E), and exhibited peering down behavior at the edges of the open arms (Figure 3F).

In contrast to the *Brinp3*^−/−^ mice, *Brinp2*^−/−^ mice did not show an increased preference for the open arms of the EPM, indicating a normal response to potential danger (Figure 3G). However, male but not female mice did show increased velocity in the test, consistent with hyperactivity (Figure 3H).

An increase in open arm exploration was also apparent in the *Brinp2/3*^−/−^ mice (Figures 3I,J), consistent with the absence of *Brinp3* alone, suggesting that this reduced anxiety phenotype is not modified by the absence of *Brinp2*.

### *Brinp2^−/−^, Brinp3^−/−^*, and *Brinp2/3^−/−^* mice exhibit normal sensory gating and short-term memory

The startle response and PPI test is used to measure sensory gating in mice, and models deficits in human subjects diagnosed with schizophrenia. *Brinp2*^−/−^*, Brinp3*^−/−^, and *Brinp2/3*^−/−^ mice were able to inhibit the startle response when primed with pre-pulses of 4, 8, and 16 dB (Supplementary Figures 4A,B). The ability of all three knock-out lines to gate their startle response indicates that these mice do not model this aspect of human schizophrenia.

**Figure 4:**
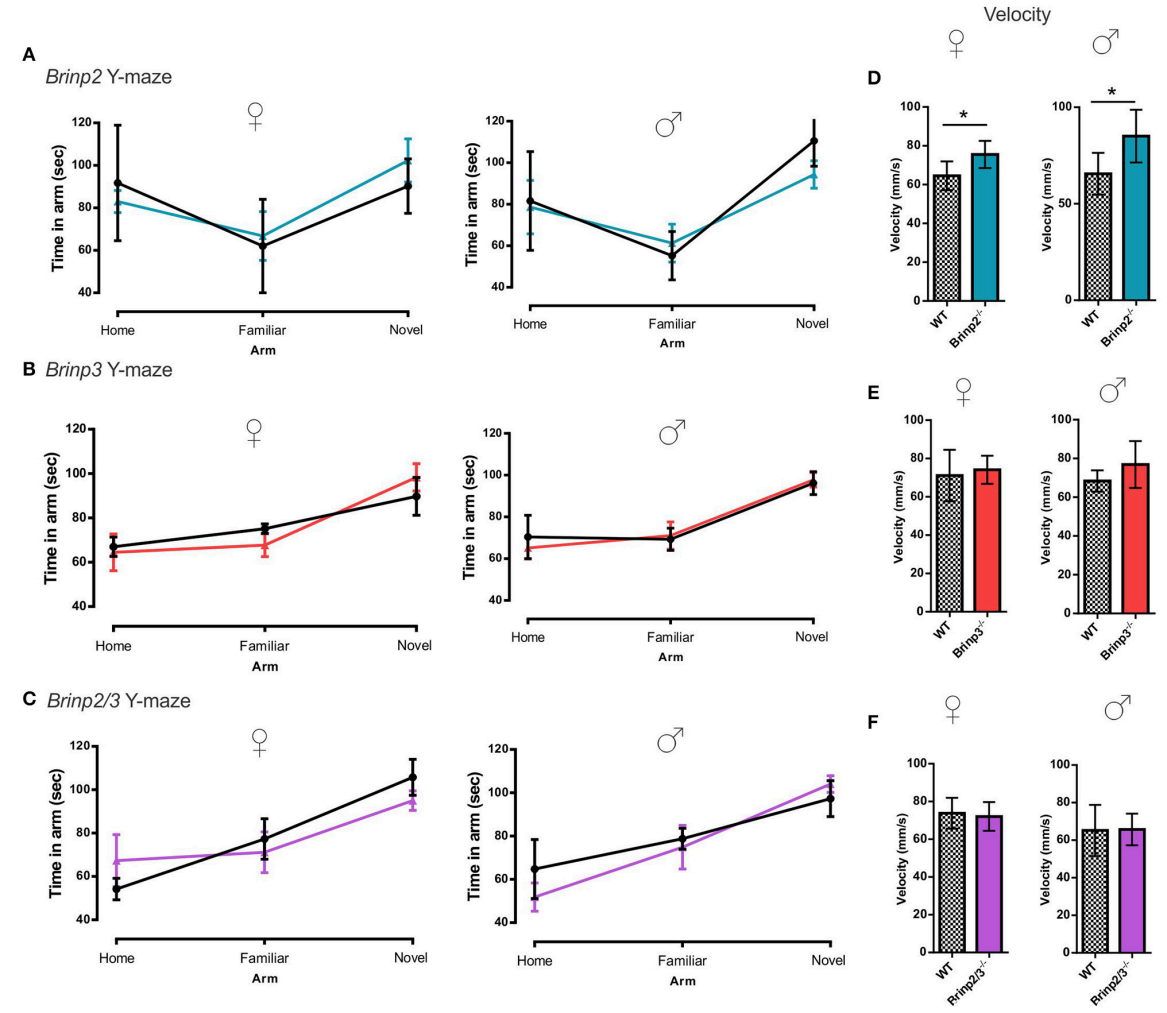
**Y-maze: normal short-term memory. (A)** *Brinp2*^−/−^ Y-maze: analysis by repeat measures two-way ANOVA shows no interaction effect between genotype × arm; female: *F*_(2, 20)_ = 0.842, *p* = 0.446, male: *F*_(2, 20)_ = 1.521, *p* = 0.243. *N* = 6 female, 6 male mice per genotype. **(B)** *Brinp3*^−/−^ Y-maze: No interaction effect between genotype × arm; female: *F*_(2, 16)_ = 0.582, *p* = 0.574, male: *F*_(2, 24)_ = 0.136, *p* = 0.874. *N* = 5 female, 7 male mice per genotype, two-way repeat measures ANOVA. **(C)** *Brinp2/3*^−/−^ Y-maze: No interaction effect between genotype × arm; female: *F*_(2, 20)_ = 0.791, *p* = 0.467, male: *F*_(2, 20)_ = 0.864, *p* = 0.436. *N* = 6 female, 6 male mice per genotype, two-way repeat measures ANOVA. **(D)** *Brinp2*^−/−^ mice show increased average velocity during Y-maze testing, female: *t*_(10)_ = 2.637, *p* = 0.025, male: *t*_(10)_ = 2.504, *p* = 0.037, Student's *t*-test. **(E)** Normal *Brinp3*^−/−^ mice average velocity during Y-maze testing, female: *t*_(8)_ = 0.435, *p* = 0.675, male: *t*_(12)_ = 1.694, *p* = 0.116, Student's *t*-test. **(F)** Normal *Brinp2/3*^−/−^ mice average velocity during Y-maze testing, female: *t*_(10)_ = 0.385 *p* = 0.709 male: *t*_(10)_ = 0.082, *p* = 0.937, Student's *t*-test.^*^p < 0.05, Data presented as the mean ± *SD*.

*Brinp2*^−/−^*, Brinp3*^−/−^, and *Brinp2/3*^−/−^ mice were tested for spatial learning and memory in the Y-maze. Following a 2-h interval after exploring the home and a single arm of the maze, mice were recorded exploring the three arms of the maze. As expected, WT control mice for each line preferred to explore the novel arm in this test. All three knock-out lines tested also showed the species-typical significant increase in time spent exploring the novel arm compared to the familiar arm, indicating normal short-term memory (Figures 4A–C). *Brinp2*^−/−^ mice moved at a statistically significantly increased velocity for this test (Figure 4D), consistent with hyperactivity, whereas *Brinp3*^−/−^ and *Brinp2/3*^−/−^ mice did not show a significant increase in locomotor activity (Figures 4E,F).

### *Brinp3^−/−^* and *Brinp2/3^−/−^* mice exhibit a mild reduction in sociability; *Brinp2^−/−^* mice show increased locomotor activity

**Figure 5:**
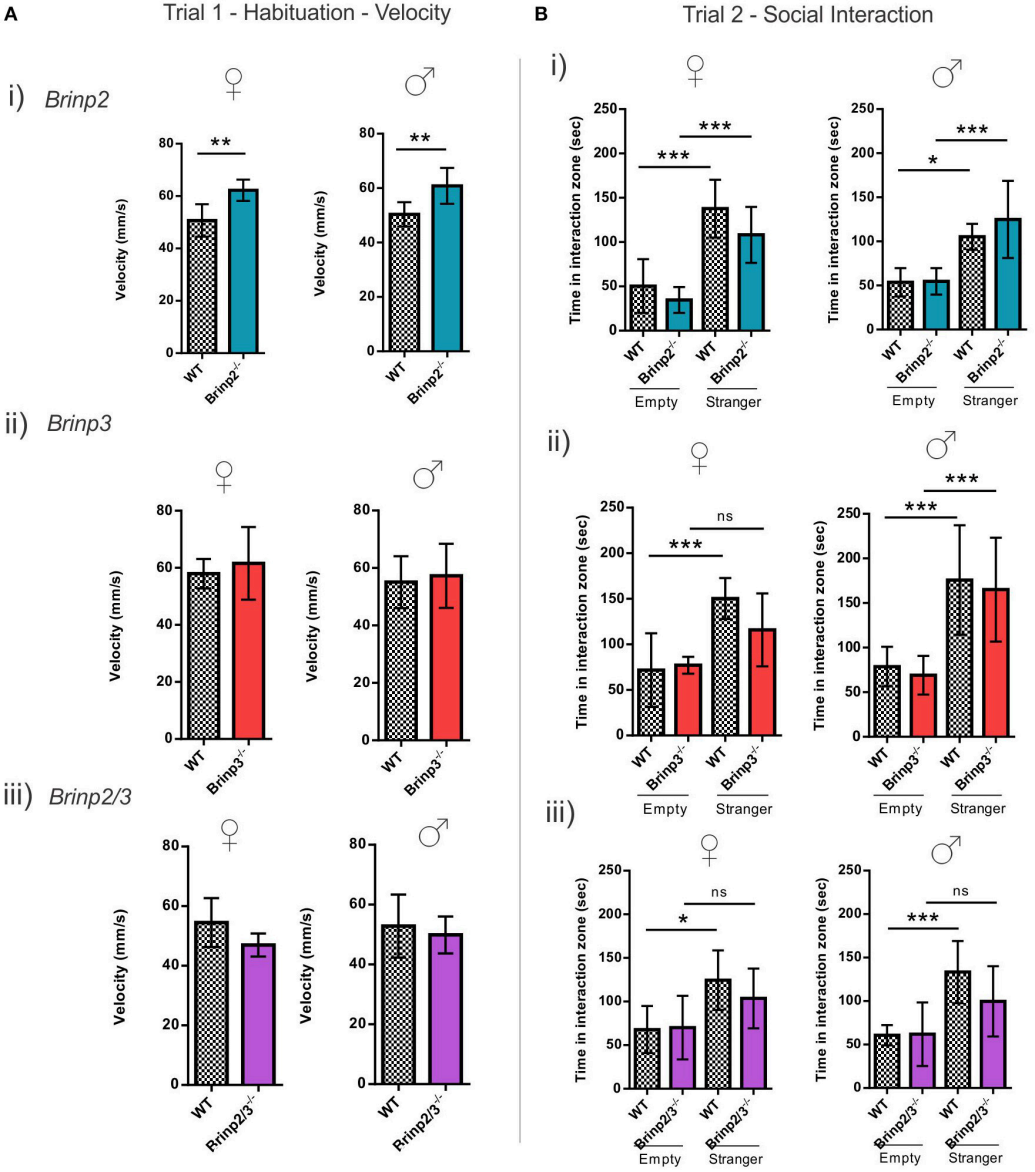
**Social interaction of ***Brinp2***^**−/−**^, ***Brinp3***^**−/−**^, and ***Brinp2/3***^**−/−**^ knock-out mice. (A)** Velocity Trial 1: (i) *Brinp2*^−/−^ mice exhibited increased average velocity over 10 min trial interval whilst habituating to the area in trial 1, indicating hyperactivity. Female: *p* = 0.0035, Male: *p* = 0.0091, Student's *t*-test. (ii) and (iii) *Brinp3*^−/−^ and *Brinp2/3*^−/−^ showed normal activity (velocity) during the habituation trial. *Brinp3*^−/−^ female: *p* = 0.5708 *Brinp3*^−/−^ male: *p* = 0.6920, *Brinp2/3*^−/−^ female: *p* = 0.0938 *Brinp2/3*^−/−^ male: *p* = 0.5956, Student's *t*-test. **(B)** Social Interaction Trial 2: (i) *Brinp2*^−/−^ mice display the expected significant increase in interaction time with the stranger mouse compared to an empty cage, indicating normal sociability: Female: *F*_(3, 23)_ = 17.598, *p* < 0.001, male: *F*_(3, 22)_ = 8.310, *p* < 0.001, one way ANOVA. Tukey HSD *post-hoc* test: Female: WT empty—WT stranger: *p* < 0.001, *Brinp2*^−/−^ empty—*Brinp2*^−/−^ stranger: *p* = 0.001, WT empty—*Brinp2*^−/−^ empty: *p* = 0.772, WT stranger—*Brinp2*^−/−^ stranger: *p* = 0.297. Male: WT empty—WT stranger: *p* = 0.033, *Brinp2*^−/−^ empty—*Brinp2*^−/−^ stranger: *p* = 0.001, WT empty—*Brinp2*^−/−^ empty: *p* = 1.000, WT stranger—*Brinp2*^−/−^ stranger: *p* = 0.582, *N* = 6 females, 6 males per genotype. (ii) *Brinp3*^−/−^ (female only) mice do not show a significant increase in interaction time with the stranger mouse compared to an empty cage: *Brinp3*^−/−^ female: *F*_(3, 19)_ = 7.057, *p* = 0.003, male: *F*_(3, 27)_ = 10.868, *P* < 0.001, one way ANOVA. Tukey HSD *post-hoc* test: Female: WT empty—WT stranger: *p* = 0.005, *Brinp3*^−/−^ empty—*Brinp3*^−/−^ stranger: *p* = 0.233, WT empty—*Brinp3*^−/−^ empty: *p* = 0.993, WT stranger—*Brinp3*^−/−^ stranger: *p* = 0.329. Male: WT empty—WT stranger: *p* = 0.003, *Brinp3*^−/−^ empty—*Brinp3*^−/−^ stranger: *p* = 0.003, WT empty—*Brinp3*^−/−^ empty: *p* = 0.977, WT stranger—*Brinp3*^−/−^ stranger: *p* = 0.969, *N* = 5 females, 7 males per genotype. (iii) *Brinp2/3*^−/−^ mice do not show a significant increase in interaction time with the stranger mouse compared to an empty cage: *Brinp2/3*^−/−^ female: *F*_(3, 21)_ = 3.977, *p* = 0.025, male: *F*_(3, 23)_ = 6.606, *p* = 0.003, one way ANOVA. Tukey HSD *post-hoc* test: Female: WT empty—WT stranger: *p* = 0.036, *Brinp2/3*^−/−^ empty—*Brinp2/3*^−/−^ stranger: *p* = 0.399, WT empty—*Brinp2/3*^−/−^ empty: *p* = 0.999, WT stranger—*Brinp2/3*^−/−^ stranger: *p* = 0.722. Male: WT empty—WT stranger: *p* = 0.006, *Brinp2/3*^−/−^ empty—*Brinp2/3*^−/−^ stranger: *p* = 0.229, WT empty—*Brinp2/3*^−/−^ empty: *p* = 1.000, WT stranger—*Brinp2/3*^−/−^ stranger: *p* = 0.321, *N* = 6 females, 6 males per genotype. ns = not significant. ^*^p < 0.05, ^**^p < 0.01, ^***^p < 0.001. Data presented as the mean ± *SD*.

The three chamber social interaction test is routinely used to investigate sociability in rodents (Silverman et al., 2010). In the habituation trial (trial 1) mice were allowed to explore the whole arena, including left and right chambers containing empty cages. *Brinp2*^−/−^*, Brinp3*^−/−^, and *Brinp2/3*^−/−^ mice spent a normal amount of time exploring the cages, without preference for left or right chambers (Supplementary Figure 5A). *Brinp2*^−/−^ mice exhibited increased velocity in this trial (Figure 5A), resulting an increased total distance traveled (Supplementary Figure 5B). Neither *Brinp3*^−/−^ nor *Brinp2/3*^−/−^ mice showed significant increases in velocity/distance traveled (Figure 5A and Supplementary Figure 5B).

Whilst hyperactive, *Brinp2*^−/−^ mice exhibited normal sociability in this test. In contrast, *Brinp3*^−/−^ females and *Brinp2/3*^−/−^ mice of both sexes did not show the expected increase in interaction time when investigating the cage containing a stranger mouse compared to an empty cage (Figure 5B). No significant difference was detected between WT and knock-outs (*Brinp3*^−/−^ and *Brinp2/3*^−/−^) in the interaction time with the stranger mouse. Overall, these finding suggest that absence of *Brinp3* reduces sociability.

## Discussion

In this study we have found that absence of *Brinp2* or *Brinp3* alone or in combination has no overt effect on mouse anatomy, reproduction, or viability, but has an age-specific negative effect on bodyweight. Knockout mice exhibit behavioral traits consistent with NDDs, including hyper-exploration, hyperactivity and reduced sociability.

### *Brinp3^−/−^* mice exhibit hyper-exploratory behavior

The reduced aversion of *Brinp3*^−/−^ mice to the open arms of the Elevated Plus Maze demonstrates these mice have an altered response to potential danger: specifically, that the *Brinp3*^−/−^ anxiety response is lessened. A similar but less pronounced phenotype is exhibited by *Brinp1*^−/−^ mice (Kobayashi et al., 2014; Berkowicz et al., 2016). The fact that both *Brinp1*^−/−^ and *Brinp3*^−/−^ mice exhibit this phenotype, along with sociability changes, may indicate a role for both genes in regulating anxiety as well as sociability. Consistent with this suggestion, both *Brinp1* and *Brinp3* are highly expressed in the adult amygdala complex, a brain region governing fear and other emotions (Prather et al., 2001; Kalin et al., 2004).

The repetitive peering down behavior of *Brinp3*^−/−^ mice has been previously reported in the dopamine transporter (DAT knock-out) mouse model for ADHD as the “cliff avoidance reaction” (Yamashita et al., 2013), which could indicate that changes in synaptic dopamine levels may contribute to this behavior in *Brinp3*^−/−^ mice. Another explanation could be changes in levels of the stress-response neurotransmitter norepinephrine in the *Brinp3*^−/−^ mice, as *Brinp3* expression correlates with changes in norepinephrine levels (Goddard et al., 2010; Karoly et al., 2012). Future steps to investigate underlying physiology, as well as the use of fear response paradigms, such as fear avoidance and fear conditioning, may further elucidate the role of *Brinp3* in relation to human anxiety disorders.

### Increased locomotor activity of *Brinp2^−/−^* mice

*Brinp2*^−/−^ mice exhibit increased locomotor activity, a phenotype also observed in *Brinp1*^−/−^ mice (Kobayashi et al., 2014; Berkowicz et al., 2016). This hyperactivity may model the reported ADHD in patients with alterations at the 1q25.2 locus (Lesch et al., 2008; Romanos et al., 2008; Lionel et al., 2014). To determine whether these mice show face validity for human ADHD, the *Brinp2*^−/−^ mice would require testing for attention and impulsivity as key diagnostic criteria. The 5-choice serial reaction time test is an ideal approach, as this paradigm has been established for assessing both attention and impulse control in rodents (Higgins and Breysse, 2008; Asinof and Paine, 2014).

*Brinp2*^−/−^ mice show a reduction in body weight as adults (males only). This reduced body mass may reflect increased energy expenditure due to hyperactivity. The enhanced Rotarod performance by female *Brinp2*^−/−^ mice may also be due to increased locomotor activity, as reported for other hyperactive mice (Graham and Sidhu, 2010; Bohuslavova et al., 2016). It is intriguing that *Brinp2*^−/−^ mice are hyperactive, whilst *Brinp2/3*^−/−^ mice are not. Perhaps the absence of *Brinp3* results in behavior that reduces overall locomotor activity. For example, frequent peering-down along the edges of the open arms of the EPM would reduce overall horizontal plane velocity.

### *Brinp2/3^−/−^* mice resemble *Brinp3^−/−^* mice

The phenotype of *Brinp2/3*^−/−^ mice shows a high degree of similarity to that of the *Brinp3*^−/−^mice. The weight profile is almost identical, as is the hyper-exploratory phenotype detected on the elevated plus maze, normal velocity in various tests, and the changes in sociability. These results suggest that apart perhaps from locomotor activity, there is no significant phenotypic alteration or enhancement in the double knock-out mice compared to the single knock-out mice. Therefore, the genes do not compensate to mask phenotypic behaviors of the single knock-out mice.

*Brinp2 and Brinp3* show partial overlap in expression profile during development, including co-expression in brain regions that include the cerebellum, neocortex, and olfactory bulb (Kawano et al., 2004). It is however notable that there are regions where *Brinp2 and Brinp3* show distinct expression profiles, e.g., *Brinp2* is highly expressed in the CA1, CA2, and CA3 regions of the adult hippocampus, whilst *Brinp3* is predominantly expressed in the dentate gyrus. *Brinp3* also shows broader expression in the cerebellum (Kawano et al., 2004). Taken with our findings, this suggests that either *Brinp2*/*Brinp3* have distinct molecular functions, or *Brinp2* and *Brinp3* carry out the same role, but function in distinct neuronal subtypes.

### Comparison with the *Brinp1^−/−^* mice phenotype

Comparing *Brinp2*^−/−^ and *Brinp3*^−/−^ mice phenotypes to previously reported *Brinp1*^−/−^ mice (Kobayashi et al., 2014; Berkowicz et al., 2016), it is evident that there is some areas of overlap, but overall mice do not show the same breadth of severity as the *Brinp1*^−/−^ mice. For instance, neither *Brinp2*^−/−^ or *Brinp3*^−/−^ mice exhibit reduced viability, impaired short-term memory and do not show the same degree of reduced sociability or decreased body weight. This is consistent with the observation that *Brinp1* is the most highly and ubiquitously expressed of the three genes during brain development (Kawano et al., 2004). The overlap in some, but not all phenotypes between mice lacking *Brinp2*^−/−^ or *Brinp3*^−/−^ with the *Brinp1*^−/−^ *mice* indicates possible shared *Brinp1*–*Brinp2* and *Brinp1*–*Brinp3* molecular functions.

### *Brinp1/2/3^−/−^* mice: initial insights into mice lacking all *Brinps*

The poor survival of litters from *Brinp1/2/3* heterozygous breeders is likely related to the absence of *Brinp1*, as we have previously reported (Berkowicz et al., 2016). Although the additional absence of *Brinp2* and *Brinp3* likely exacerbates the phenotype, as appears to be the case when viability of litters drops further when male mice of genotype *Brinp1*^−/+^*2*^−/−^*3*^−/−^ are bred with female triple-het mice. This drop in viability of litters above that of litters heterozygous for all three *Brinp* genes may be due to the cumulative effect of the absence of *Brinp3 and Brinp1* in reducing sociability, and therefore affecting parental care of offspring. Altogether, a different breeding strategy, or a much larger breeding colony of triple-hets is needed for generating sufficient numbers of triple KO out mice for a behavioral testing cohort of 10 mice of a similar age.

The survival past weaning of some *Brinp1/2/3*^−/−^ triple knock-out mice indicates that *Brinps* are not essential for embryonic or neonatal development. The absence of all three *Brinps* results in an ostensibly normal, but significantly smaller mouse, with normal gross morphology of all neural and non-neural tissue, implying that *Brinps* are not essential for organogenesis or neurogenesis. The normal gross anatomy of the *Brinp1/2/3*^−/−^ mice is surprising given the high expression of Brinps in overlapping regions during murine neural development (Kawano et al., 2004). A greater level of histopathological analysis, using layer specific markers, is needed to determine whether *Brinp1/2/3*^−/−^ mice have altered neural architecture.

The reduction in body weight of *Brinp1/2/3*^−/−^ mice may reflect the observed phenotype of *Brinp1*^−/−^ mice. Male *Brinp1/2/3*^−/−^ mice especially are significantly smaller from even the *Brinp1*^−/−^ mice (Berkowicz et al., 2016). This may be the additive effect of the reduced body weight of *Brinp1*^−/−^ and *Brinp2*^−/−^ mice, both of which weigh less than WT as adults.

## Concluding remarks

This study is a first step in understanding the effect of *Brinp2* and *Brinp3* genes on cognitive function. The observed changes in *Brinp3*^−/−^ anxiety response, and increased *Brinp2*^−/−^ locomotor activity may be relevant to the genetic variation at the 1q25.2 locus associated with patients diagnosed with ADHD, anxiety and other NDDs. *Brinp2*^−/−^ and *Brinp3*^−/−^ genes do not appear to compensate in the mammalian brain and are therefore likely have distinct molecular or cell specific functions.

## Author contributions

PB and JW conceived the study. SB and PB designed and co-ordinated the study. SB carried out knock-out mouse validation, mouse reproductive and weight analysis, statistical analysis, compiled the figures. SB drafted the manuscript with revision by PB. TF performed behavioral testing of mice. All authors reviewed and approved the final manuscript.

### Conflict of interest statement

The authors declare that the research was conducted in the absence of any commercial or financial relationships that could be construed as a potential conflict of interest.

## Supplementary material

Supplementary Figure 1 **Reduced litter survival when breeding Triple-het and Triple-het × *Brinp1*^−/+^*2*^−/−^*3*^−/−^ mice**. Breeders were monitored for litter size at birth and litter size at age of weaning (P21). **(A)** No significant differences in number of pups per litter at postnatal day 0, from WT/WT, Triple-Het/Triple-Het and Triple-Het ♀/*Brinp1*^−/+^*2*^−/−^*3*^−/−^ ♂ parents, *F*_(2, 35)_ = 2.702, *p* = 0.0811, one-way ANOVA. **(B)** The combined *Brinp1, Brinp2*, and *Brinp3* deleted allele of breeders impacted the number of pups weaned at postnatal day 21, from WT/WT, Triple-Het/ Triple-Het and Triple-Het ♀/*Brinp1*^−/+^*2*^−/−^*3*^−/−^ ♂ parents. *F*_(2, 35)_ = 7.142, p<0.0025, one-way ANOVA. Tukey HSD multiple comparisons tests showed significant differences: WT × WT and Triple-Het × Triple-Het: *p* = 0.842, WT × WT and Triple-Het × *Brinp1*^−/+^*2*^−/−^*3*^−/−^: *p* = 0.0025, Triple-Het × Triple-Het and Triple-Het × *Brinp1*^−/+^*2*^−/−^*3*^−/−^: *p* = 0.027.^*^*p* < 0.05, ^**^*p* < 0.001, *N* = 3 breeding pairs per genotype, 10–14 litters per genotype.

Supplementary Figure 2 ***Vision and Olfaction*. (A)** Normal vision for *Brinp2*^−/−^, *Brinp3*^−/−^ and *Brinp2/3*^−/−^ mice: (i) *Brinp2*^−/−^ Vision test female: *t*_(10)_ = 0.674, *p* = 0.516 male: *t*_(10)_ = 0.277, *p* = 0.787, Student's *t*-test. (ii) *Brinp3* Vision test female: *t*_(8)_ = 1.265, *p* = 0.242 male: *t*_(12)_ = 0.000, *p* > 0.999, Student's *t*-test. (iii) Brinp2/3 Vision test female: *t*_(9)_ = 0.302, *p* = 0.770 male: *t*_(10)_ = 0.568, *p* = 0.583, Student's *t*-test. **(B)** Normal olfaction for *Brinp2*^−/−^ and *Brinp3*^−/−^ mice: (i) Brinp2 Olfaction female: *F*_(1, 10)_ = 1.773, *p* = 0.213, male: *F*_(1, 10)_ = 0.081, *p* = 0.781, repeat measures two-way ANOVA. (ii) Brinp3 Olfaction female: *F*_(1, 8)_ = 1.513, *p* = 0.254, male: *F*_(1, 12)_ = 0.538, *p* = 0.477, repeat measures two-way ANOVA. Data presented as the mean ± *SD*.

Supplementary Figure 3 **Rotarod. (A)** Female *Brinp2*^−/−^ mice show significant improvement in latency to fall on the Rotarod; female: *F*_(1, 10)_ = 10.464, *p* = 0.009, male: *F*_(1, 10)_ = 0.769, *p* = 0.401, repeat measures two-way ANOVA. *N* = 6 female, 6 male mice per genotype. **(B)** *Brinp3*^−/−^ mice do not show significant motor co-ordination impairment on the Rotarod; female: *F*_(1, 8)_ = 1.337, *p* = 0.281, male *F*_(1, 12)_ = 1.483, *p* = 0.247, repeat measures two-way ANOVA. *N* = 5 female, 7 male mice per genotype. **(C)** *Brinp2/3*^−/−^ mice do not show significant motor co-ordination impairment on the Rotarod; female: *F*_(1, 8)_ = 1.560, *p* = 0.247, male *F*_(1, 10)_ = 0.087, *p* = 0.774, repeat measures two-way ANOVA. N = 6 female, 6 male mice per genotype. Data presented as the mean ± *SD*.

Supplementary Figure 4 **Startle and Pre-pulse Inhibition (PPI). (A)** Normal startle response for *Brinp2, Brinp3*, and *Brinp2/3* mice: (i) *Brinp2*^−/−^ startle female: *t*_(9)_ = 0.1178, *p* = 0.908, male: *t*_(10)_ = 0.4818, *p* = 0.640, Student's *t*-test. (ii) *Brinp3*^−/−^ startle female: *t*_(8)_ = 0.6158, *p* = 0.555, male: *t*_(12)_ = 0.2630, *p* = 0.797, Student's *t*-test. (iii) *Brinp2/3*^−/−^ startle female: *t*_(9)_ = 0.7216, *p* = 0.489, male: *t*_(10)_ = 0.2195, *p* = 0.831, Student's *t*-test. **(B)** Normal Pre Pulse Inhibition (PPI) for *Brinp2*^−/−^, *Brinp3*^−/−^, and *Brinp2/3*^−/−^ mice: (i) *Brinp2*^−/−^ PPI female: *F*_(1, 10)_ = 3.551, *p* = 0.089, male: *F*_(1, 10)_ = 0.783, *p* = 0.397, repeat measures two-way ANOVA. (ii) *Brinp3*^−/−^ PPI female: *F*_(1, 8)_ = 0.276, *p* = 0.614, male: *F*_(1, 12)_ = 0.056, *p* = 0.817, repeat measures two-way ANOVA. (iii) *Brinp2/3*^−/−^ PPI female: *F*_(1, 9)_ = 0.056, *p* = 0.818, male: *F*_(1, 10)_ = 0.133, *p* = 0.753, repeat measures two-way ANOVA. Data presented as the mean ± *SD*.

Supplementary Figure 5 **Habituation trial of three-chamber social interaction test (Trial 1). (A)** Habituation trial of the three chamber social interaction test, showing interaction time between empty cages. No significant preference between the left/right chambers for *Brinp2*^−/−^, *Brinp3*^−/−^, or *Brinp2/3*^−/−^ mice. (i) *Brinp2*^−/−^ SI Test Trial 1: female: *F*_(3, 23_ = 0.371, *p* = 0.775, male: *F*_(3, 23)_ = 0.119, *p* = 0.948, one-way ANOVA, N = 6 females, 6 males per genotype. (ii) *Brinp3*^−/−^ SI Test Trial 1: female: *F*_(3, 19)_ = 0.399, *p* = 0.756, male: *F*_(3, 27)_ = 1.144, *p* = 0.352, one-way ANOVA, N = 5 females, 7 males per genotype. (iii) *Brinp2/3*^−/−^ SI Test Trial 1: female: *F*_(3, 23)_ = 0.621, *p* = 0.609, male: *F*_(3, 21)_ = 0.086, *p* = 0.967, one-way ANOVA, N = 6 females, 6 males per genotype. **(B)** Distance Travelled Trial 1. (i) *Brinp2*^−/−^ mice traveled a significantly increased distance over 10 min trial interval whilst habituating to the arena, indicating hyperactivity. Female: *p* = 0.0163, male: *p* = 0.0061, Student's *t*-test. (ii–iii) *Brinp3*^−/−^ and *Brinp2/3*^−/−^ showed normal activity (distance traveled) during the habituation trial. *Brinp3*^−/−^ female: *p* = 0.5199, *Brinp3*^−/−^ male: *p* = 0.6300, *Brinp2/3*^−/−^ female: *p* = 0.5030, *Brinp2/3*^−/−^ male: *p* = 0.4608, Student's *t*-test.
