# Supplementary figures and images for: Mice Lacking Brinp2 or Brinp3, or Both, Exhibit Behaviors Consistent with Neurodevelopmental Disorders
Source: Front Behav Neurosci. 2016 Oct 25;10:196. doi: 10.3389/fnbeh.2016.00196 (PMC5079073; doi:10.3389/fnbeh.2016.00196)

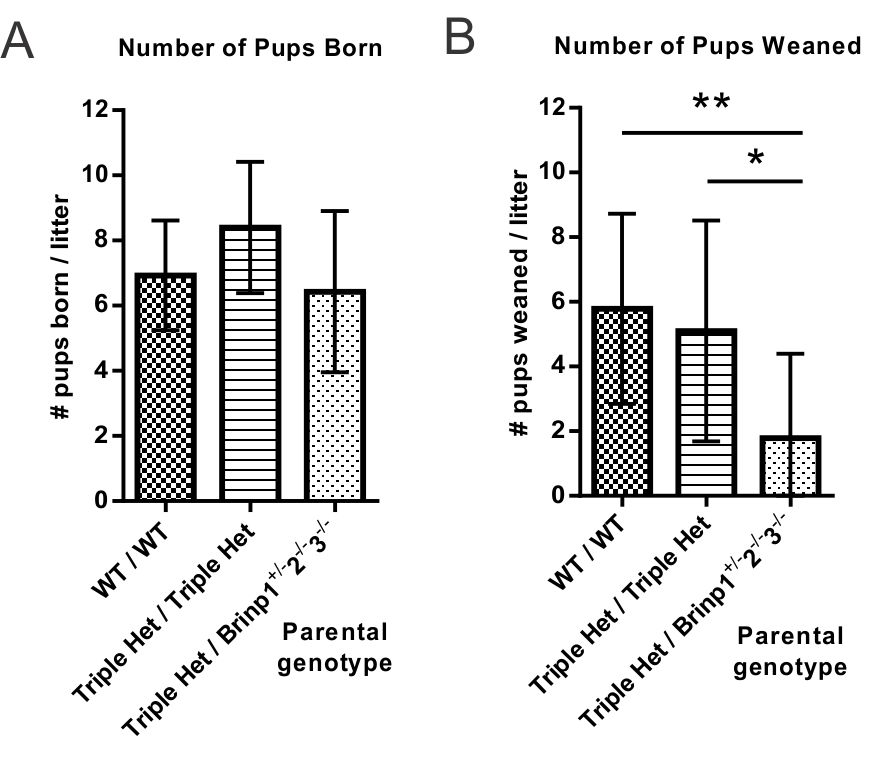

Supplement: Supplementary Figure 1 — Reduced litter survival when breeding Triple-het and Triple-het × Brinp1−/+2−/−3−/− mice. Breeders were monitored for litter size at birth and litter size at age of weaning (P21). (A) No significant differences in number of pups per litter at postnatal day 0, from WT/WT, Triple-Het/Triple-Het and Triple-Het ♀/Brinp1−/+2−/−3−/− ♂ parents, F(2, 35) = 2.702, p = 0.0811, one-way ANOVA. (B) The combined Brinp1, Brinp2, and Brinp3 deleted allele of breeders impacted the number of pups weaned at postnatal day 21, from WT/WT, Triple-Het/ Triple-Het and Triple-Het ♀/Brinp1−/+2−/−3−/− ♂ parents. F(2, 35) = 7.142, p<0.0025, one-way ANOVA. Tukey HSD multiple comparisons tests showed significant differences: WT × WT and Triple-Het × Triple-Het: p = 0.842, WT × WT and Triple-Het × Brinp1−/+2−/−3−/−: p = 0.0025, Triple-Het × Triple-Het and Triple-Het × Brinp1−/+2−/−3−/−: p = 0.027.*p < 0.05, **p < 0.001, N = 3 breeding pairs per genotype, 10–14 litters per genotype. [file Image1.JPEG]

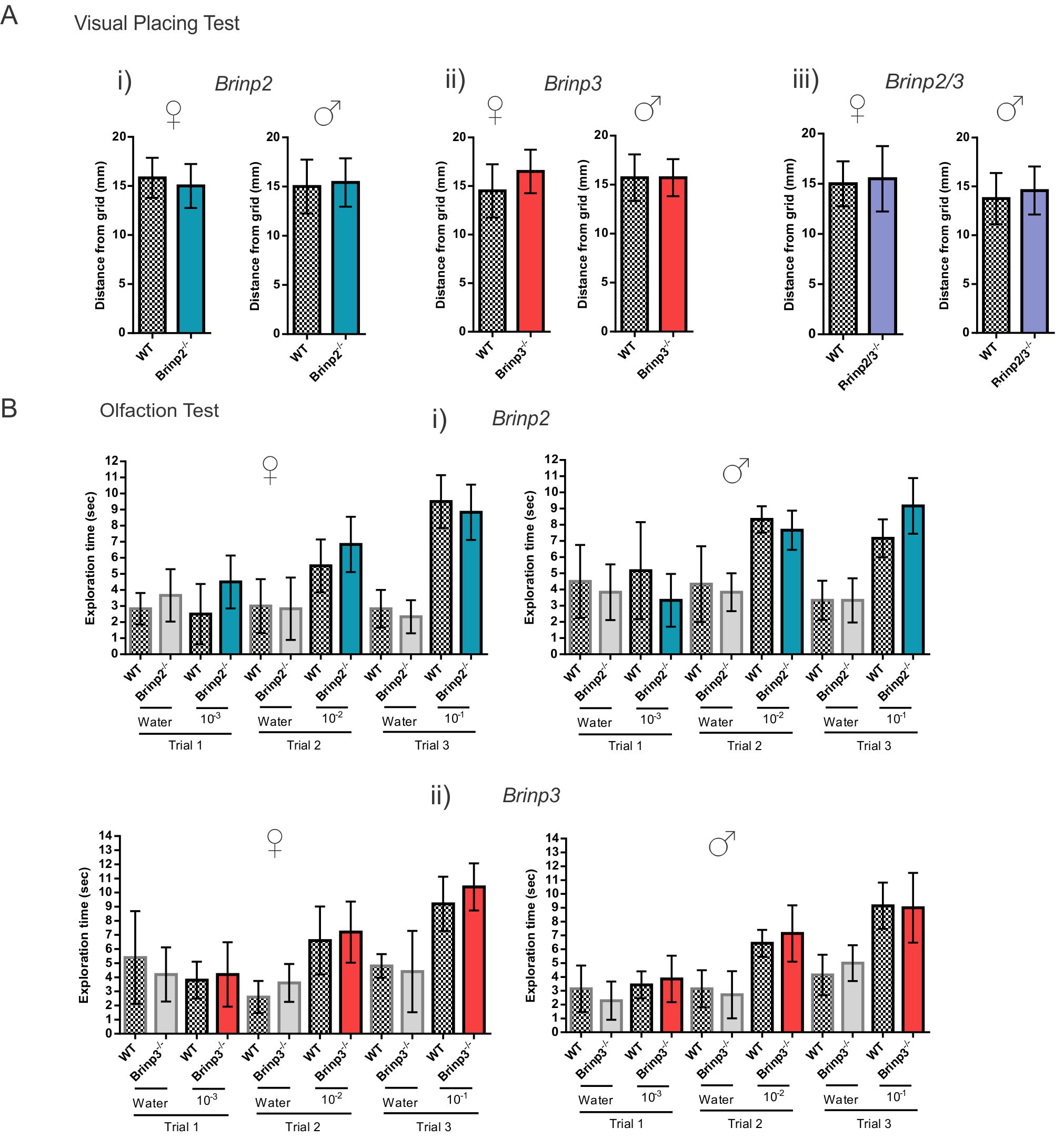

Supplement: Supplementary Figure 2 — Vision and Olfaction. (A) Normal vision for Brinp2−/−, Brinp3−/− and Brinp2/3−/− mice: (i) Brinp2−/− Vision test female: t(10) = 0.674, p = 0.516 male: t(10) = 0.277, p = 0.787, Student's t-test. (ii) Brinp3 Vision test female: t(8) = 1.265, p = 0.242 male: t(12) = 0.000, p > 0.999, Student's t-test. (iii) Brinp2/3 Vision test female: t(9) = 0.302, p = 0.770 male: t(10) = 0.568, p = 0.583, Student's t-test. (B) Normal olfaction for Brinp2−/− and Brinp3−/− mice: (i) Brinp2 Olfaction female: F(1, 10) = 1.773, p = 0.213, male: F(1, 10) = 0.081, p = 0.781, repeat measures two-way ANOVA. (ii) Brinp3 Olfaction female: F(1, 8) = 1.513, p = 0.254, male: F(1, 12) = 0.538, p = 0.477, repeat measures two-way ANOVA. Data presented as the mean ± SD. [file Image2.jpg]

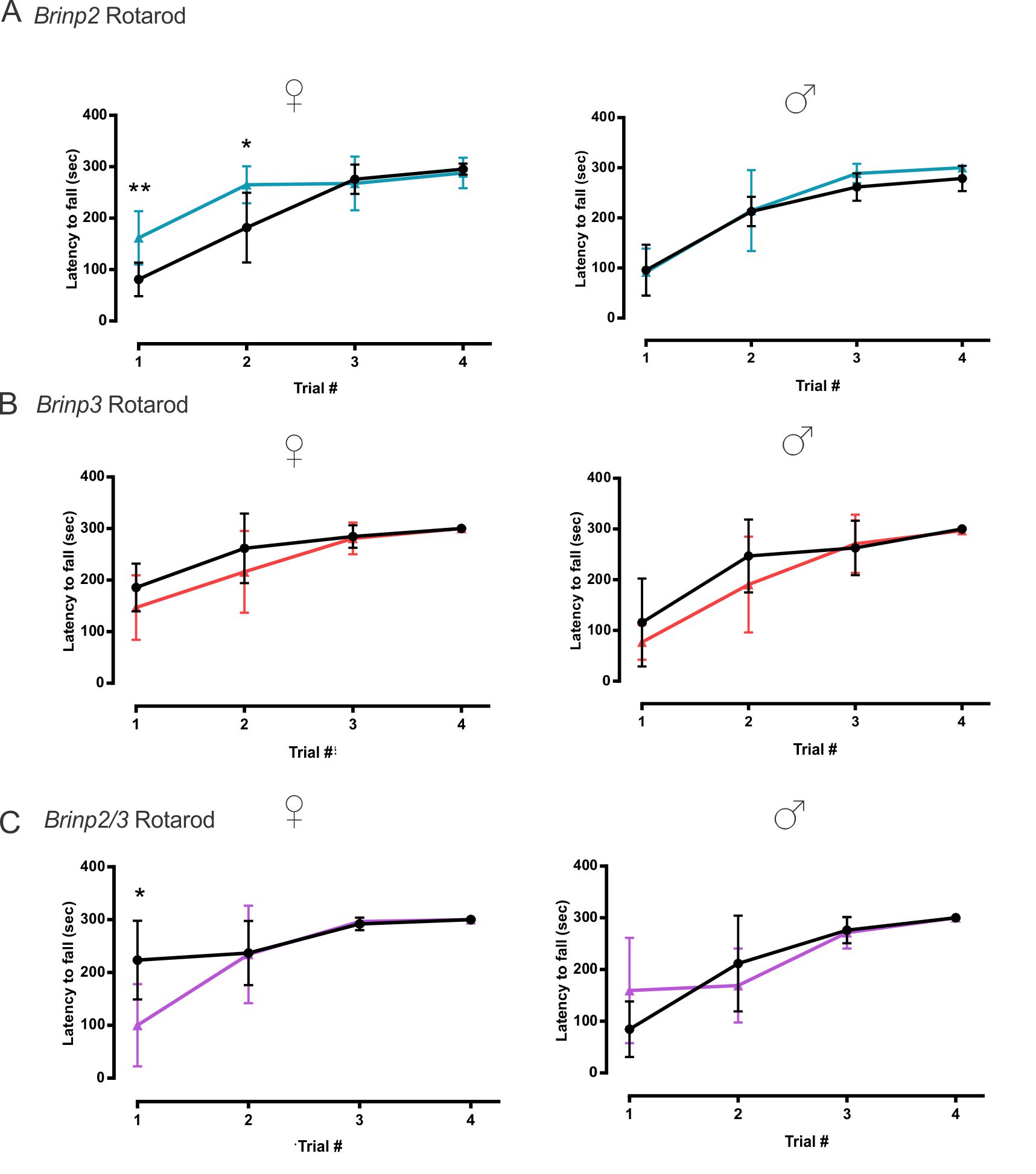

Supplement: Supplementary Figure 3 — Rotarod. (A) Female Brinp2−/− mice show significant improvement in latency to fall on the Rotarod; female: F(1, 10) = 10.464, p = 0.009, male: F(1, 10) = 0.769, p = 0.401, repeat measures two-way ANOVA. N = 6 female, 6 male mice per genotype. (B) Brinp3−/− mice do not show significant motor co-ordination impairment on the Rotarod; female: F(1, 8) = 1.337, p = 0.281, male F(1, 12) = 1.483, p = 0.247, repeat measures two-way ANOVA. N = 5 female, 7 male mice per genotype. (C) Brinp2/3−/− mice do not show significant motor co-ordination impairment on the Rotarod; female: F(1, 8) = 1.560, p = 0.247, male F(1, 10) = 0.087, p = 0.774, repeat measures two-way ANOVA. N = 6 female, 6 male mice per genotype. Data presented as the mean ± SD. [file Image3.JPEG]

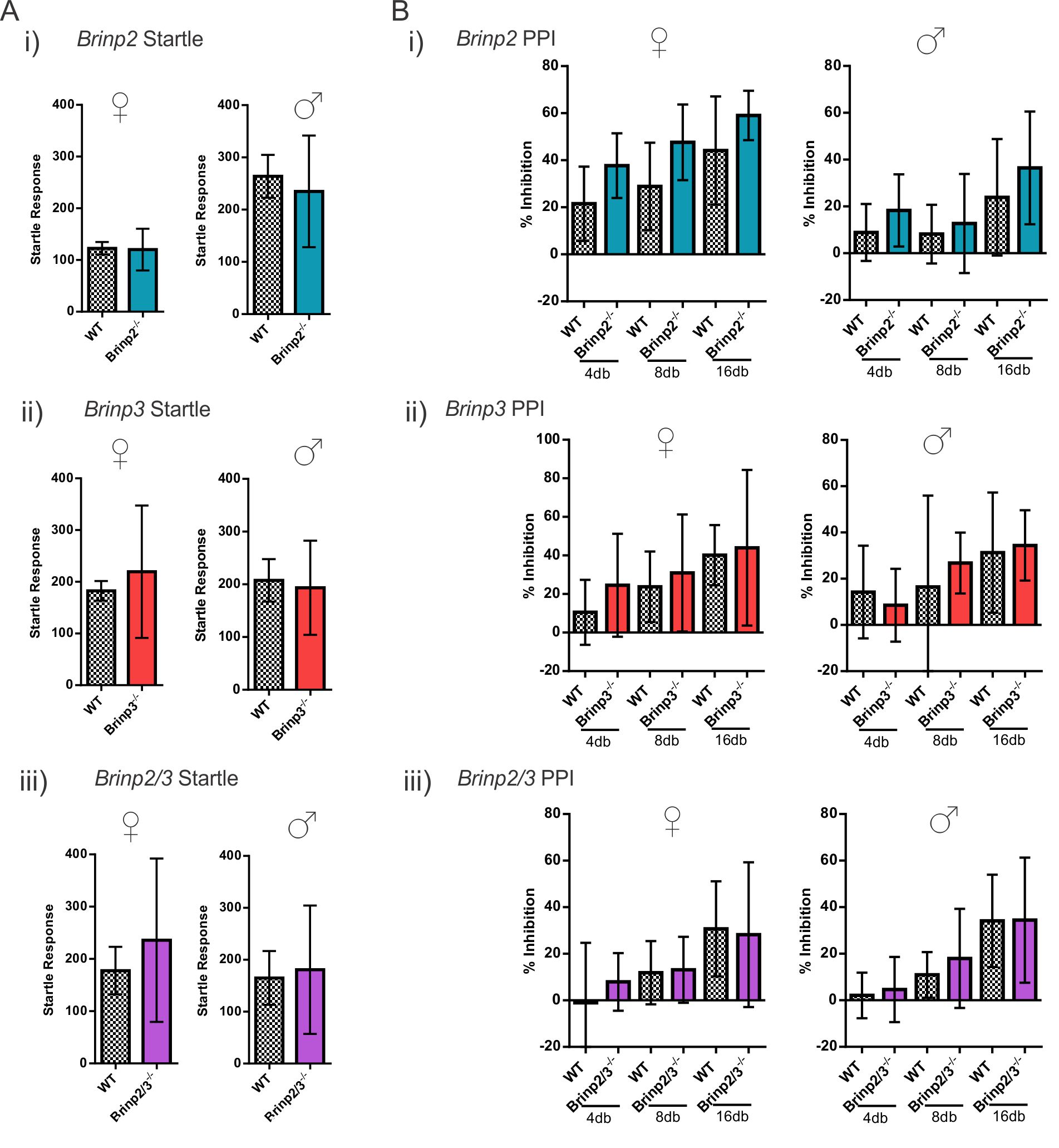

Supplement: Supplementary Figure 4 — Startle and Pre-pulse Inhibition (PPI). (A) Normal startle response for Brinp2, Brinp3, and Brinp2/3 mice: (i) Brinp2−/− startle female: t(9) = 0.1178, p = 0.908, male: t(10) = 0.4818, p = 0.640, Student's t-test. (ii) Brinp3−/− startle female: t(8) = 0.6158, p = 0.555, male: t(12) = 0.2630, p = 0.797, Student's t-test. (iii) Brinp2/3−/− startle female: t(9) = 0.7216, p = 0.489, male: t(10) = 0.2195, p = 0.831, Student's t-test. (B) Normal Pre Pulse Inhibition (PPI) for Brinp2−/−, Brinp3−/−, and Brinp2/3−/− mice: (i) Brinp2−/− PPI female: F(1, 10) = 3.551, p = 0.089, male: F(1, 10) = 0.783, p = 0.397, repeat measures two-way ANOVA. (ii) Brinp3−/− PPI female: F(1, 8) = 0.276, p = 0.614, male: F(1, 12) = 0.056, p = 0.817, repeat measures two-way ANOVA. (iii) Brinp2/3−/− PPI female: F(1, 9) = 0.056, p = 0.818, male: F(1, 10) = 0.133, p = 0.753, repeat measures two-way ANOVA. Data presented as the mean ± SD. [file Image4.jpg]

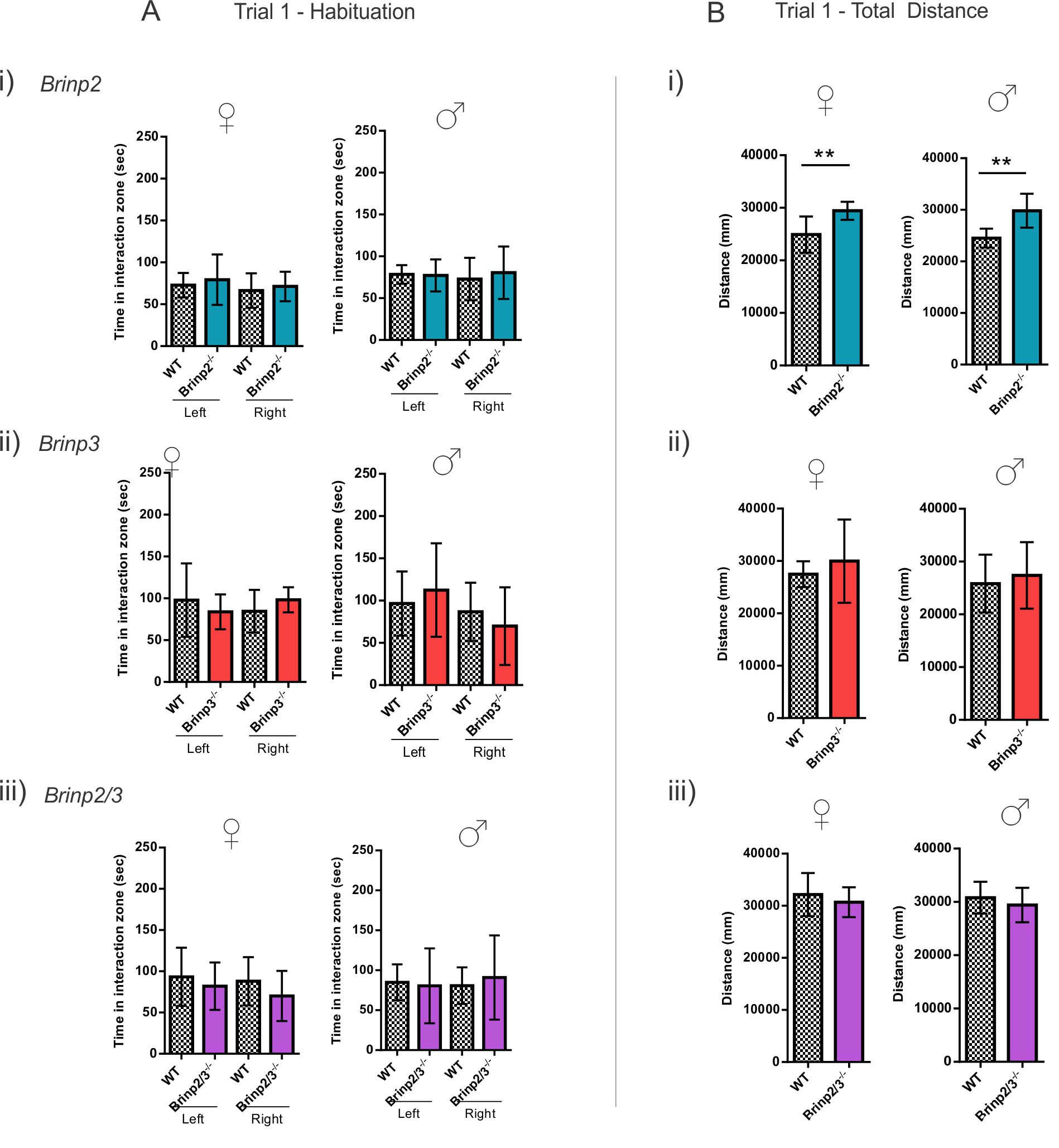

Supplement: Supplementary Figure 5 — Habituation trial of three-chamber social interaction test (Trial 1). (A) Habituation trial of the three chamber social interaction test, showing interaction time between empty cages. No significant preference between the left/right chambers for Brinp2−/−, Brinp3−/−, or Brinp2/3−/− mice. (i) Brinp2−/− SI Test Trial 1: female: F(3, 23 = 0.371, p = 0.775, male: F(3, 23) = 0.119, p = 0.948, one-way ANOVA, N = 6 females, 6 males per genotype. (ii) Brinp3−/− SI Test Trial 1: female: F(3, 19) = 0.399, p = 0.756, male: F(3, 27) = 1.144, p = 0.352, one-way ANOVA, N = 5 females, 7 males per genotype. (iii) Brinp2/3−/− SI Test Trial 1: female: F(3, 23) = 0.621, p = 0.609, male: F(3, 21) = 0.086, p = 0.967, one-way ANOVA, N = 6 females, 6 males per genotype. (B) Distance Travelled Trial 1. (i) Brinp2−/− mice traveled a significantly increased distance over 10 min trial interval whilst habituating to the arena, indicating hyperactivity. Female: p = 0.0163, male: p = 0.0061, Student's t-test. (ii–iii) Brinp3−/− and Brinp2/3−/− showed normal activity (distance traveled) during the habituation trial. Brinp3−/− female: p = 0.5199, Brinp3−/− male: p = 0.6300, Brinp2/3−/− female: p = 0.5030, Brinp2/3−/− male: p = 0.4608, Student's t-test. [file Image5.JPEG]
